# Epitranscriptomic control of stress adaptations in *Escherichia coli*

**DOI:** 10.1093/nar/gkag042

**Published:** 2026-02-02

**Authors:** Sebastián Riquelme-Barrios, Siobhan A Cusack, Luis Rivera-Montero, Leonardo Vásquez-Camus, Korinna Burdack, Sophie Brameyer, Maximilian Berg, G Nur Yeşiltaç-Tosun, Stefanie Kaiser, Pascal Giehr, Kirsten Jung

**Affiliations:** Faculty of Biology, Microbiology, Ludwig-Maximilians-Universität München, Martinsried 82152, Germany; Faculty of Biology, Microbiology, Ludwig-Maximilians-Universität München, Martinsried 82152, Germany; Faculty of Biology, Microbiology, Ludwig-Maximilians-Universität München, Martinsried 82152, Germany; Faculty of Biology, Microbiology, Ludwig-Maximilians-Universität München, Martinsried 82152, Germany; Faculty of Biology, Microbiology, Ludwig-Maximilians-Universität München, Martinsried 82152, Germany; Faculty of Biology, Microbiology, Ludwig-Maximilians-Universität München, Martinsried 82152, Germany; Goethe University Frankfurt, Faculty 14, Institute of Pharmaceutical Chemistry, Frankfurt 60438, Germany; Goethe University Frankfurt, Faculty 14, Institute of Pharmaceutical Chemistry, Frankfurt 60438, Germany; Goethe University Frankfurt, Faculty 14, Institute of Pharmaceutical Chemistry, Frankfurt 60438, Germany; Department of Chemistry, Ludwig-Maximilians-Universität München, München 81377, Germany; Faculty of Biology, Microbiology, Ludwig-Maximilians-Universität München, Martinsried 82152, Germany

## Abstract

The impacts of various stressors on bacterial systems have been studied at the phenotypic, transcriptional, and translational levels during the early stress response. However, the contributions of RNA modifications during stress adaptation remain largely unexplored. Here, we map the epitranscriptomic changes of *Escherichia coli* after exposure to oxidative and acid stress using direct RNA sequencing of mRNA, rRNA, pre-tRNA, and tRNA, combined with mass spectrometry, deletion mutant phenotyping, and single-nucleotide PCR. We identified widespread, dynamic mRNA modifications that include central metabolism transcripts and increased levels of rRNA methylations (m^4^Cm and m^5^C) under both stresses, with potential consequences for translation. In uncharged pre-tRNAs, stress-specific modifications via the Mnm and Q pathways accumulated at the wobble position; these modifications proved crucial for survival. Together, these findings reveal a multifaceted layer of post-transcriptional regulation, establishing the first comprehensive view of the bacterial epitranscriptome during the early stress response.

## Graphical Abstract

**Graphical Abstract:**
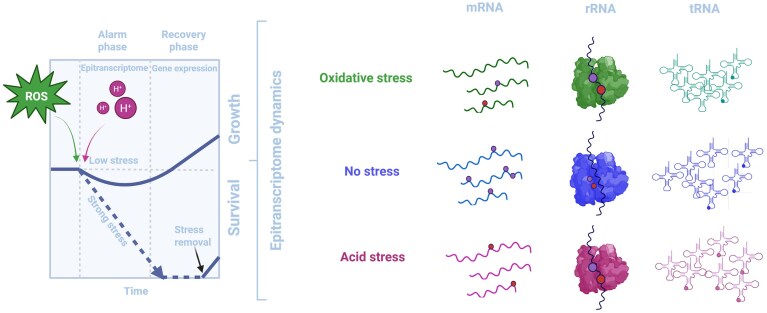

## Introduction

**Figure 1.**
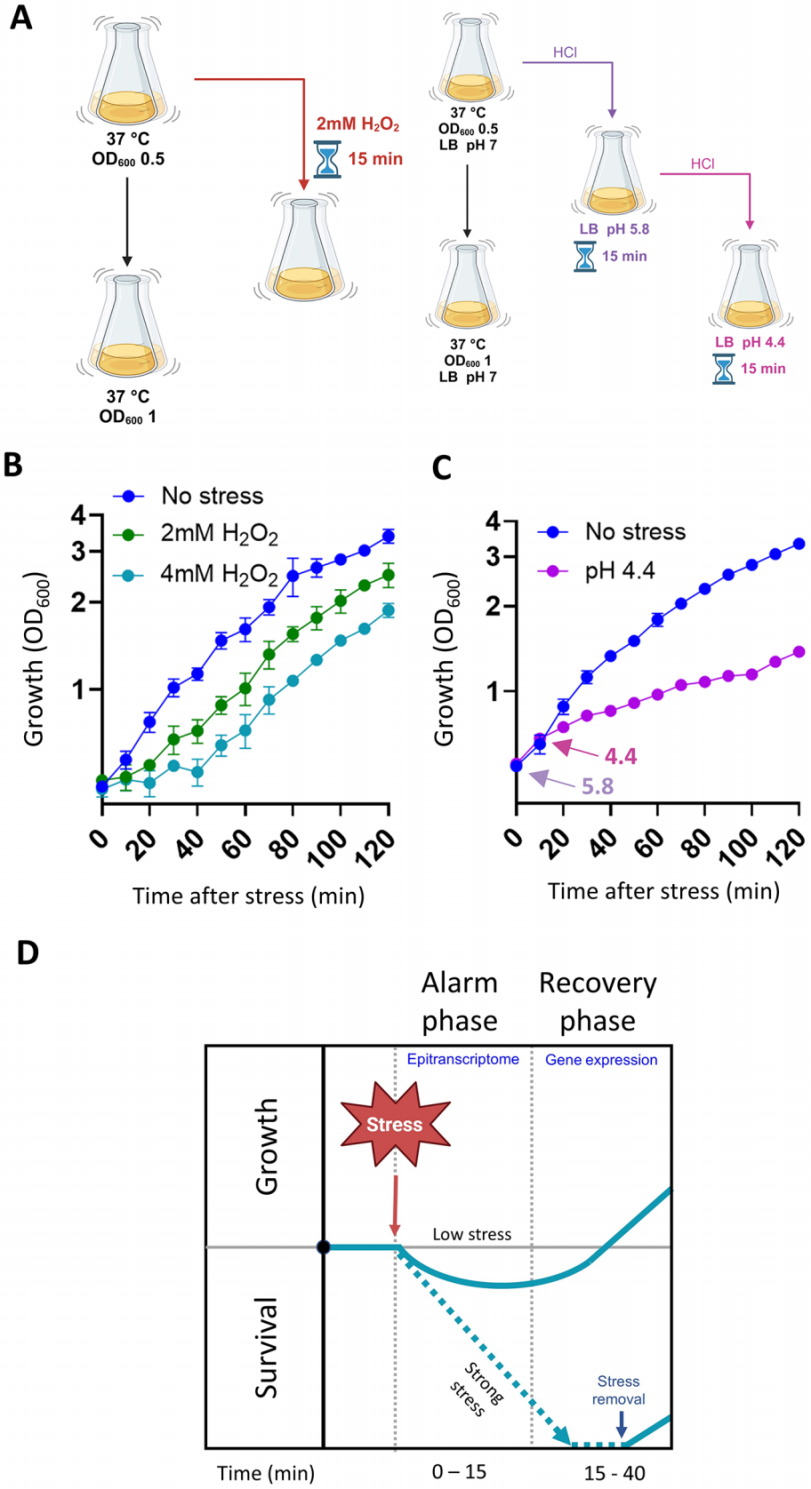
The early stress response in *E. coli* is characterized by decreased and delayed growth. (**A**) Overview of the oxidative and acid stress protocols used in the present study. For each stress condition, two flasks of *E. coli* were grown in parallel at 37°C to an OD_600_ of 0.5. For oxidative stress, H_2_O_2_ was added to one flask to a final concentration of 2 mM, and cultivation was continued at 37°C for 15 min. For acid stress, HCl was added to one flask to decrease the pH to 5.8. Cultivation was then continued at 37°C for 15 min, after which additional HCl was added to reach pH 4.4. For both stress conditions, the untreated flask remained incubating at 37°C. Cells were then harvested, and RNA was extracted. Created in BioRender. https://BioRender.com/bfqb68z. (**B**) Growth of control (no stress) *E. coli* and those exposed to 2 or 4 mM H_2_O_2_ over time from 0–120 min post-stress. OD_600_ was used as a measure of bacterial growth. (**C**) Growth of control (no stress) *E. coli* and those grown in pH 4.4 media over time from 0–120 min post-stress. Arrows indicate the initial shift to mild acid stress (pH 5.8) and the subsequent shift to severe acid stress (pH 4.4). OD_600_ was used as a measure of bacterial growth. (**D**) Illustration of the bacterial stress response. The first component of the early stress response is the alarm phase, during which an adverse condition is sensed, and growth slows or halts. In the case of a relatively mild stress, bacteria proceed to the recovery phase, during which remodeling of gene expression takes place and growth resumes. Under strong stress, the bacteria die or survive in a dormant state, from which they can continue to grow once the stimulus is removed.

Bacteria are often subjected to environmental stresses, including sudden changes in pH, temperature, osmolarity, nutrient availability, or reactive oxygen species levels. Across biological systems [1–6], the early phase of the stress response, known as the alarm phase, involves stress sensing, during which adverse external conditions are identified and transduced via signaling processes. Growth is usually halted to redirect resources toward recovery. The latter part of the alarm phase overlaps with the recovery phase, which involves cellular changes at multiple levels (e.g. transcriptomic, translational, and proteomic) to adapt to stress and resume growth (Fig. 1D).

Modifications of bacterial tRNAs and rRNAs have been characterized in detail, with roles in structure, catalytic activity, assembly, maturation, translation fidelity, tRNA structure, and codon recognition [7–14]. *In vitro* studies have shown that modifications in mRNAs can impair codon–anticodon interactions, destabilize the ribosome, and increase amino acid substitution errors, impacting reading accuracy at stop codons [15–17]. Previous work from our group demonstrated RNA modifications in *E. coli* that vary between growth stages [18]. More recently, our group identified putative modification sites in *E. coli* mRNAs that exhibit altered abundance in response to heat stress [19]. Here, we studied the epitranscriptome after exposure of *E. coli* to severe acid or oxidative stress. These conditions are biologically relevant to strains of enteropathogenic *E. coli* (which are exposed to severe acid stress during passage through the stomach [20]) and uropathogenic *E. coli* (which are exposed to high levels of host-generated reactive oxygen species in the bladder [21]).

The roles of some epitranscriptomic marks during bacterial stress responses have been characterized, particularly in rRNAs. For example, C1402 of the decoding center in the 16S rRNA is modified by both RsmI (which forms Cm) and RsmH [22] (m^4^C), together forming *N*^4^,2′-*O*-dimethylcytidine (m^4^Cm); these enzymes are implicated in virulence and oxidative stress resistance in *Staphylococcus aureus* [23]. Levels of 5-hydroxycytidine (ho^5^C) at C2501 of the *E. coli* 23S rRNA increase during oxidative stress, impairing protein synthesis and thus conferring a protective effect [24].

In addition to the rRNA, the roles of some bacterial tRNA modifications throughout the stress response have been defined. In *Pseudomonas aeruginosa*, tRNA methylation (by members of the Trm family) is necessary for resistance to oxidative stress and is involved in survival during infection. Specifically, the absence of *trmJ* increases sensitivity to H_2_O_2_ [25], and Δ*trmB* mutants have impaired expression of mRNAs enriched in Phe and Asp codons, such as *katB* and *katA* [26]. Similarly, mutants for *ttcA*, which catalyzes post-transcriptional thiolation of C32 (s^2^C32), show H_2_O_2_ hypersensitivity and attenuated infection capacity [27]. Overall, H_2_O_2_ exposure produces an increase in 7-methylguanosine (m^7^G) levels; relatedly, a lack of functional *trmB* produces an oxidative stress-sensitive phenotype in *P. aeruginosa* [26]. The lack of MnmA, which catalyzes 2-thiouridine formation [28], shows hypersensitivity to H_2_O_2_ (1 mM) [29]. In response to acid stress, one *E. coli* screen identified the tRNA modification gene *mnmE* as important for growth [30]. A *Streptococcus mutans* mutant lacking *gidA* (a homolog of the *E. coli* modification gene *mnmG*, which, together with *mnmE*, is responsible for 5-methylaminomethyl-2-thiouridine [mnm^5^s^2^U] modifications) shows increased sensitivity to mild acid stress. Δ*mnmG* and Δ*mnmE* mutants also have difficulties adapting to temperature and osmotic stresses [31]. A similar trend was observed in *Cronobacter sakazakii*, where transposon mutagenesis of *mnmG* causes increased acid sensitivity [32].

Some stress conditions alter modifications in the wobble position of the tRNA anticodon specifically. These modifications change the binding preference of the anticodon, promoting non-Watson–Crick interactions with the corresponding codon. In some cases, codons that require a modification in the wobble position for translation (i.e. modification-dependent codons) are more abundant among mRNAs that are expressed in response to stress or specific metabolic signals. Conditions that increase wobble-position modification levels can thus initiate translation of transcripts containing a high abundance of modification-dependent codons [33] in a regulatory mechanism referred to as modification tunable transcripts (MoTTs) [34, 35]. Although some instances of MoTTs regulation have been described in *E. coli*, few stress-responsive MoTTs have been identified in this organism [36].

Despite the numerous insights yielded by prior studies of tRNA and rRNA modifications under stress conditions, a comprehensive analysis of the *E. coli* epitranscriptomic response to oxidative or acid stress during the early stress response has not yet been conducted. To uncover specific and general epitranscriptomic responses to stress conditions during this phase, we conducted a multifaceted analysis incorporating direct RNA sequencing (DRS), mass spectrometry (MS), single-nucleotide PCR, and mutant phenotype characterization. This allowed us to assess changes in the abundance of mRNA, pre-tRNA, tRNA, and rRNA modifications in response to stress, revealing both specific and unique stress responses.

## Material and methods

### Strains and growth conditions

For experiments conducted with the wild-type (WT) *E. coli* strain MG1655, bacteria were cultured in lysogeny broth (LB) with 200 rpm shaking at 37°C to an optical density at 600 nm (OD_600_) of 0.5. No-stress control cells were kept for 30 min at 37°C to an OD_600_ of ∼0.8–1. For oxidative stress, 3% H_2_O_2_ was added to a final concentration of 2 or 4 mM in the bacterial culture. For acid stress, 5 M HCl was added to reduce the pH of the growth medium from 7 to 5.8. After 15 min, additional HCl was added to reduce the pH to 4.4. For both stress conditions, bacteria were grown at 37°C for an additional 15 min after treatment before sample collection [5]. Strains carrying plasmids were grown overnight with chloramphenicol; the antibiotic was omitted during the stress experiment.

The single mutants ∆*rsmH*, ∆*rsmI*, ∆*rsmA*, ∆*rsmJ*, ∆*mnmA*, ∆*mnmE*, and ∆*tgt* were generated via in-frame deletion as previously described [19]. Primers used for mutant construction are listed in Supplementary Table S1. ∆*rsmF* mutant strains were generated for a prior publication [37]. For complementation of ∆*rsmF*, the gene was inserted back into the *E. coli* genome in the original position with in-frame replacement. For complementation of ∆*rsmH*, ∆*mnmA*, ∆*mnmE*, and ∆*tgt*, the corresponding gene and its native promoter region were inserted into the *pBAD33* plasmid via Gibson assembly after linearization using two primers to eliminate the arabinose promoter (Supplementary Table S1).

### Total RNA isolation, tRNA/rRNA depletion, and mRNA enrichment

RNA was isolated using the PCI protocol [38] with some modifications [18]. To produce mRNA-enriched (mRNAe) samples for nanopore sequencing, the resulting total RNA was treated to remove tRNAs and deplete ribosomes as previously described [19]. Primers for reverse transcription (RT)-quantitative PCR (qPCR) to verify ribosome depletion are shown in Supplementary Table S1. For MS analysis, RNA samples were separated using a size-selection protocol following the manufacturer’s instructions (RNA Clean & Concentrator Kit, Zymo Research, Irvine, CA, USA). The resulting > 200 nt fraction was used to assess rRNA modifications, and the ≤200 nt fraction was used to evaluate tRNA modifications. Sample purity and size were confirmed via electrophoresis using the 2100 Bioanalyzer (Agilent Technologies, Santa Clara, CA, USA).

### RT-qPCR

cDNA was reverse transcribed from purified RNA samples, and RT was carried out for qPCR to quantify expression of *katG, cadB, rrlH* (23S rRNA), *rrsH* (16S rRNA), and *recA* using previously described methods [19]. Gene expression was calculated as the abundance of the gene of interest normalized to that of the 16S rRNA using the X^–ΔCt^ method, where X corresponds to the primer-pair-specific efficiency, which was experimentally calculated from serially diluted DNA template. These values were 1.94 (*cadB*), 1.96 (*rrsH* and *katG*), and 2 (*rrlH* and *recA*). RT-qPCR primer sequences are shown in Supplementary Table S1.

### MS analysis of RNA modifications

Absolute quantitative analyses using isotope dilution MS were performed as previously described [19] with the addition of N4-acetylcytidine (ac^4^C) and aminocarboxypropyluridine (acp^3^U) (Carbosynth, Staad, Switzerland); Q (provided by the Peter Dedon laboratory); and 5-methylaminomethyl-2-thiouridine (mnm^5^s^2^U) (provided by the Susanne Häußler laboratory). A list of monitored mass transitions is given in Supplementary Table S2.

### Nanopore sequencing

*In vitro* transcribed (IVT) RNA control samples were prepared from no-stress samples as previously described [19], with the addition of transfer-messenger RNA (tmRNA) depletion using custom probes supplied by siTOOLS (Planegg, Germany) as the final step. For mRNA-enriched samples and IVT controls, library preparation was carried out following the manufacturer’s protocol (Oxford Nanopore Technologies [ONT] #SQK-RNA004). DRS was conducted with the FLO-MIN004RA (RNA) platform (ONT, Oxford, UK) using one flow cell per sample.

### Nano-tRNAseq

Total RNA samples were extracted, purified, and treated with DNase as previously described [19]. A total of 10 µg of RNA was used to isolate the tRNA fraction (≤200 nt) through size-selection protocols following the manufacturer’s instructions (RNA Clean & Concentrator Kit, Zymo Research, Irvine, CA, USA). RNA quality and successful size selection were confirmed by electrophoresis using the 2100 Bioanalyzer (Agilent Technologies, Santa Clara, CA, USA). Subsequently, 500 ng of tRNA-enriched samples were used for library preparation following the Nano-tRNAseq protocol (Immagina Biotechnology, Trento, Italy), based on a previously described method [39]. The protocol includes a deacylation step followed by splint adapter hybridization and ligation. Barcoded adapters were then annealed and ligated, followed by RT. Six samples were pooled and prepared using the Nanopore library preparation kit #SQK-RNA004 (ONT, Oxford, UK) for RNA ligation adapter attachment. Finally, the libraries were loaded onto a FLO-MIN004RA (RNA) flow cell and sequenced according to the manufacturer’s protocol.

### ONT data processing

Reads from ONT sequencing were basecalled using Dorado v5.0.0 (ONT, Oxford, UK). SeqKit [40] v2.8.2 was used to calculate basic statistics, namely the average read length, N50 length, and the total read and base numbers. Average Q scores were determined with NanoPlot [41] v1.43.0. mRNAe samples were aligned with Minimap2 [42] v2.26 (using the parameters ‘-x map-ont -k14’) to a modified version of the ensemble *E. coli* reference genome (Escherichia_coli_str_k_12_substr_mg1655_gca_000005845.ASM584v2) from which duplicate tRNA and rRNA genes had been removed. Nano-tRNAseq samples were aligned to a reference file containing only *E. coli* tRNA sequences using the parameters ‘-y -t8 -ax splice -k7 -w3 -n1 -m13 -s30 -A2 -B1 -O1,32 -E1,0’, then demultiplexed by Immagina Biotechnology (Trento, Italy); demultiplexed Nano-tRNAseq reads were remapped to the tRNA reference using the parameters ‘-x map-ont -k9’. All mapped reads were filtered for quality with BamQVFilter (https://github.com/JMencius/BamQVFilter) [43] v0.1.0 at a threshold of Q score > 9 for mRNAe samples and Q score > 5 for Nano-tRNAseq samples. SAMtools [44] v1.22 was used to remove secondary and chimeric alignments and unmapped reads. The resulting quality-filtered, primary-mapped reads were classified into RNA biotypes (tRNA, rRNA, protein coding, nontranslating coding sequence [CDS], pseudogene, or ncRNA) using EnsemblBacteria annotations (GCA_000 005 845); all reads that mapped to the *ssrA* transcript were classified as tmRNA. For tRNA read length analyses, mRNAe reads classified as mapping to the tRNA were trimmed with Cutadapt v4.9[45], then the trimmed reads were mapped to the tRNA-only reference using the parameters ‘-x map-ont -k9’ and Q-score filtered as described above. Read counts were quantified using Salmon [46] v1.10.1. For differentially expressed gene (DEG) analysis, read counts from Salmon were imported into R using the ‘tximport’ package [47], and DEGs were identified using DESeq2 (v1.38.3) [48]. Significant DEGs were obtained by filtering the results based on *P*-value (≤0.05), *q*-value (≤0.05), and log_2_(fold change) (≥ 2) (Supplementary Table S3). All programs were run with default parameters unless otherwise specified.

### Putative modification site analyses

*Biotype annotations*. Putative modification sites outside the tRNA and rRNA were classified as belonging to the CDS, 5′ UTR, 3′ UTR, intergenic region, or ncRNA based on annotations in the *E. coli* K-12 substr. MG1655 ASM584v2 reference genome (RefSeq #GCF_000005845.2) and published UTR genomic coordinates [49].

*Modification detection*. Sites or regions containing a modification were identified using nanoSundial [50] with default parameters. In addition, the proportion of basecalling error (BCError) at every position of each transcript was calculated from BAM files using the “get_bcerror_freqs.py” script described by White *et al.* [51] (https://github.com/rnabioco/tRNA004/blob/main/src/modifications/get_bcerror_freqs.py). The output “BCErrorFreq” values were used in further analyses. A method of classifying sites as modified or unmodified based on BCError values was established using a combined no-stress sample (comprising biological replicates 1 and 2) and the combined IVT sample. ΔBCError was calculated for each nucleotide position as BCError_no stress_ – BCError_IVT_. Precision and recall of all known modification sites in the rRNA were calculated using a range of ΔBCError thresholds, once including and once excluding regions that were not classified as modified by nanoSundial. Based on this analysis, a ΔBCError threshold of 0.02 was used for the classification of regions in the mRNA and ncRNA as putative modification sites in the no-stress, acid-stress, and oxidative-stress samples (Supplementary Table S4).

*Modified region characterization*. The relative positions of putative modifications within a transcript were calculated based on the transcript length and the lengths of the CDS, 5′ UTR, and 3′ UTR. Modification abundance across the transcript was then visualized with the R package ‘ggplot2’ [52]. For functional annotation visualization, the custom Kyoto Encyclopedia of Genes and Genomes (KEGG) hierarchy used by Proteomaps [53] (https://www.proteomaps.net/data/KO_hierarchy.tms) was modified to include an additional ∼1000 *E. coli* genes with KEGG functional annotations (Supplementary Table S5). Transcripts and the corresponding annotations were then visualized as Voronoi treemaps with the R package ‘WeightedTreemaps’ [54].

### Modification-dependent codon abundance

Nine sets of codons were analyzed, each comprising two codons that encode the same amino acid and differ in nucleotide sequence only at the third position (Supplementary Table S6). In every set, one codon requires a wobble-position modification generated via the Q or Mnm pathway in the corresponding anticodon for translation (modification-dependent), and the other codon does not (modification-independent). The total number of all 18 codons was counted in every *E. coli* CDS using a codon usage tool (https://www.bioinformatics.org/sms2/codon_usage.html), then the normalized modification-dependent codon abundance was calculated for each as follows:


\begin{equation*}
\frac{{\# {\mathrm{\ modification\ dependent\ codons}}}}{{\# {\mathrm{\ modification\ dependent\ codons}} + \# {\mathrm{\ modification\ independent\ codons}}}}
\end{equation*}

The 400 CDSs with the highest modification-dependent codon abundance (Supplementary Table S6) were selected for further analysis. Due to a tie between the transcripts ranked in positions 399–403 for the lowest modification-dependent codon abundance, 403 transcripts were included in further analyses for this dataset (Supplementary Table S6). Functional annotations of the selected CDSs were visualized using Voronoi treemaps generated with the R package ‘WeightedTreemaps’ [54].

### Acid shock assay

Bacteria were grown in pH 7 LB medium at 37°C to an OD_600_ of 0.6–0.7. Cells were harvested via centrifugation for 2 min at 4000 × g, and cells were adjusted to an OD_600 _of 1.0 in 1 mL of fresh LB media at pH 5.8 or pH 7 as a control. After 15 min of growth at 37°C, cells were harvested again and resuspended in pH 4.4 or pH 7 LB for another 15 min of growth at 37°C. Cells were harvested one final time and resuspended in pH 3 or pH 7 LB, then grown for 1 h at 37°C. After dilution in PBS, bacteria were plated on LB agar and grown overnight at 37°C. Colony-forming units were then counted [55].

### Motility assay

Bacterial cultures were grown overnight in LB broth at 37°C. Cells were adjusted to an OD_600_ of 1.0 in 1 mL of fresh LB media. Soft agar plates [1% (w/v) tryptone, 0.25% (w/v) NaCl, and 0.3% (w/v) agar] were prepared, and 10 µL of the cell suspension was spotted at the center of each plate. Plates were incubated for 12 h at 37°C to allow bacterial growth and motility assessment. Colony size was quantified as the diameter.

### m⁵C-Rol-LAMP assay

m⁵C-Rol-LAMP was used to assess site-specific rRNA m^5^C methylation changes under pH stress [37]. Briefly, total RNA was treated for bisulfite conversion using the EZ RNA Methylation Kit (Zymo Research, Irvine, CA, USA); padlock probes were hybridized to the target sites and circularized by ligation (Supplementary Table S1). The circularized probes served as templates for rolling-circle amplification, followed by loop-mediated isothermal amplification. Reactions were performed with either dATP alone or dATP + dGTP. Signals were measured every 30 s (one measurement counted as one cycle) for 1 h using a CFX96™ Real-Time System (Bio-Rad, Hercules, CA, USA).

### Statistical analyses

Differences in the growth of mutant strains compared to the WT, in modification abundance between no-stress and acid-stress conditions (as quantified with m^5^C Rol-LAMP), and in *cadB* and *katG* expression (as quantified with RT-qPCR) were assessed with an unpaired Student’s *t*-test. A ratio-paired Student’s *t*-test was used to compare MS-based quantification of modification abundance under stress and no-stress conditions. Differences between mutant and WT cells in swimming motility and in lag time after acid shock were assessed with one-way analysis of variance with post-hoc Dunnett’s multiple comparison test. Two-way analysis of variance was used to determine statistically significant differences between mutants and WT strains with respect to doubling time and survival after acid shock; where this showed significant differences, a post-hoc Dunnett’s or Šidák correction was applied as appropriate. Differences in modification-dependent codon abundance between transcripts with significantly higher or lower translational efficiency under acid stress were assessed with a Mann–Whitney *U* test. The relationship between pre-tRNA size and cumulative BCError in the anticodon was assessed with Spearman’s rank correlation and Pearson correlation in non-normally and normally distributed data, respectively, as determined with a Shapiro–Wilk test. An α value of 0.05 was applied for all statistical tests.

## Results

### Nanopore sequencing uncovers stress-responsive epitranscriptomic signatures of mRNAs with central functions

In the present study, we sought to measure the bacterial stress response from an epitranscriptomic perspective, focusing on the early stress response during oxidative-stress (H_2_O_2_) and acid-stress (HCl) conditions (Fig. 1A, B). Stress treatment conditions were selected for their ability to perturb *E. coli* growth while ultimately allowing the bacteria to cope with the stress and resume growth. A sampling timepoint of 15 min post-stress was chosen to allow observation of the overlapping alarm and recovery phases. Oxidative stress was characterized by a dose-dependent lag phase of ∼20 and ∼35 min at 2 and 4 mM H_2_O_2_, respectively, although the growth rate remained unaffected (Fig. 1B). Under acid stress, we observed a decrease in the growth rate (Fig. 1C). These periods of decreased or halted growth are characteristic of the early stress response phase, during which bacteria redirect resources toward defense mechanisms (Fig. 1D).

**Figure 2.**
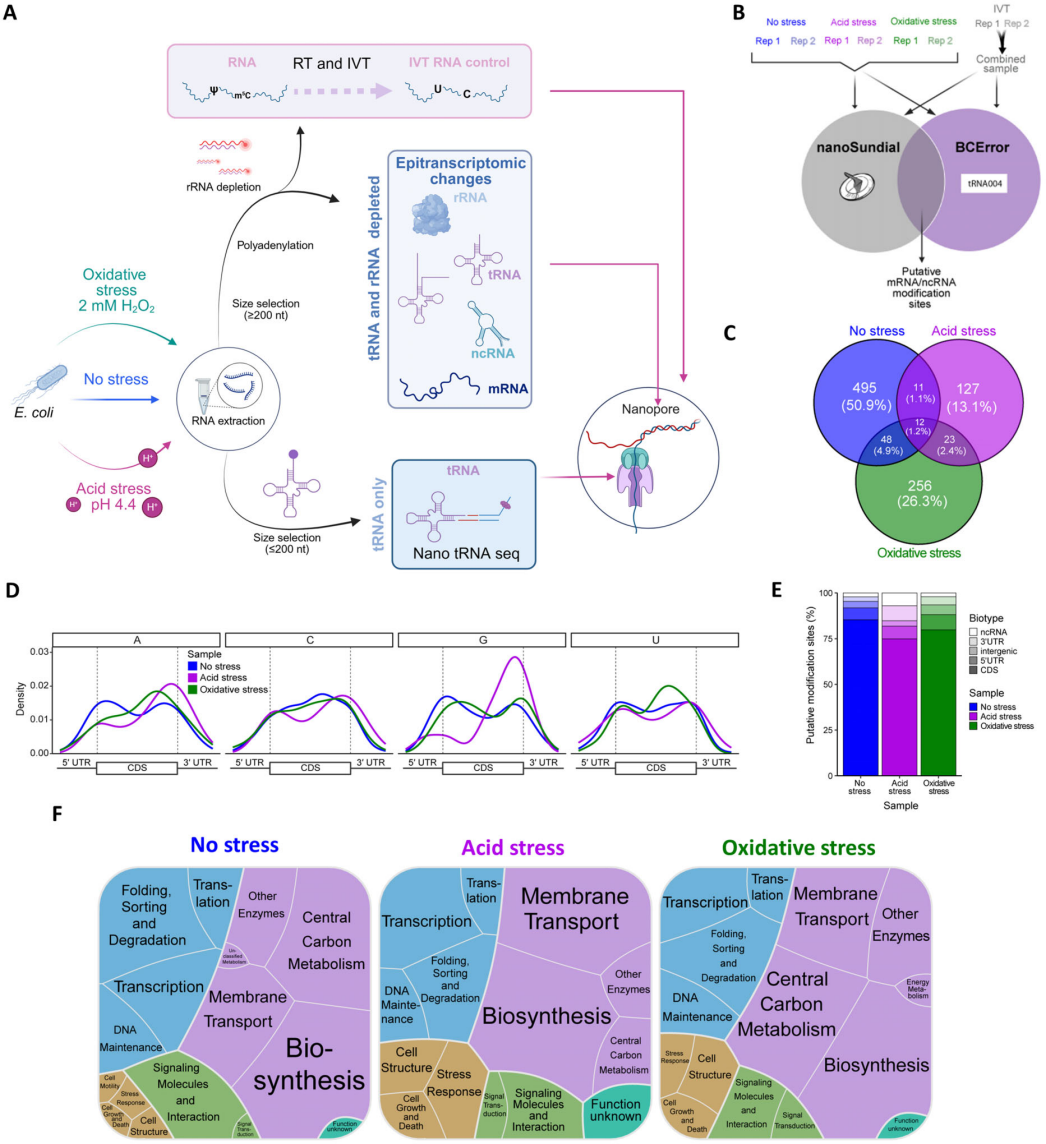
Stress-responsive epitranscriptomic signatures in mRNAs. (**A**) Schematic of the experimental design: sample treatment, library preparation, and Nanopore sequencing of RNA from control and stressed *E. coli* and production of an *in vitro* transcribed (IVT) control. For each sample type, two types of RNA preparations were produced: mRNA-enriched (mRNAe) and Nano-tRNAseq samples. tRNAs and rRNAs were depleted from the mRNAe samples, producing predominantly (but not exclusively) mRNAs and ncRNAs. In contrast, the Nano-tRNAseq samples contained only tRNAs. Created in BioRender. https://BioRender.com/k6ppi4s. (**B**) Graphical illustration of the putative novel modification detection approach. RNA was extracted from two biological replicate samples of *E. coli* grown under acid-stress, oxidative-stress, and no-stress conditions. An unmodified IVT control was generated from the RNA of no-stress samples. After direct RNA sequencing (DRS), putative modification sites outside the tRNA and rRNA were detected using basecalling error (BCError) as calculated with a publicly available script (https://github.com/rnabioco/tRNA004/blob/main/src/modifications/get_bcerror_freqs.py) and a previously published modification-detection program, nanoSundial. Modification sites/regions were considered in further analyses if they were supported by both programs in both biological replicate samples. (**C**) Summary of unique and shared putative modification sites/regions between treatment groups (no-stress, acid-stress, and oxidative-stress conditions). Most of the putative modification sites/regions detected were condition-specific, with relatively few being shared between two or all three conditions. (**D**) Distribution of putative mRNA modifications across the 5′ untranslated region (UTR), coding sequence (CDS), and 3′ UTR in each sample type. Transcript lengths were normalized to allow direct comparisons of putative modification placement. (**E**) Biotype distribution of putative novel modifications. RNA biotypes included in the analysis were the 5′ UTR, CDS, 3′ UTR, intergenic region, and ncRNA. Putative modifications were most common in the CDS across all sample conditions. (**F**) Functional annotations of transcripts containing modifications in each sample type. Cell color corresponds to the highest-level annotation for each gene as defined in the Kyoto Encyclopedia of Genes and Genomes (KEGG): Genetic Information Processing (blue), Metabolism (purple), Cell Processes (gold), Environmental Information Processing (green), or Function unknown (teal). Genes having the KEGG annotation “Function unknown” were annotated based on a literature search where possible. Cell size corresponds to the number of putative modified sites/regions present in genes with each annotation.

We next implemented DRS following previously established protocols [19, 56, 57] to analyze epitranscriptomic changes during the early stress response in no-stress controls and samples exposed to oxidative- or acid-stress conditions. A previously developed method [19] enabled simultaneous capture of rRNAs, mRNAs, ncRNAs, and uncharged pre-tRNAs in a single mRNA-enriched (mRNAe) sample (Fig. 2A; Supplementary Table S7). Additionally, we sequenced modification-free *in vitro* transcribed (IVT) RNA to control for intrinsic and sequence-specific errors (Fig. 2A). Analysis of the sequencing reads demonstrated high depth of coverage (Supplementary Fig. S1A, B), the expected CDS enrichment (Supplementary Fig. S1C), and transcriptome-level stress responses comparable to those described in previous reports [5, 49] (Supplementary Table S3; Supplementary Fig. S1D–G).

Putative modification regions were identified using two biological replicate samples per condition and two approaches for modification detection (Fig. 2B; Supplementary Fig. S2A; Supplementary Table S4). There was a low degree of overlap in putative modification regions between replicates and modification detection approaches, at 1.6% of sites in the no-stress samples (Supplementary Fig. S2B–D). A similar phenomenon was observed in the inter-condition comparison, with just 1.2% of modifications found across all three conditions (Fig. 2C). mRNA modifications were largely found in the CDS and concentrated around the start and stop codons in both the no-stress and acid-stress conditions (Fig. 2D, E). Notably, acid stress produced a strong bias in the location of modified G nucleotides, which were overwhelmingly present toward the end of the CDS under these conditions. In contrast, modifications under oxidative stress showed enrichment throughout the CDS at C nucleotides and were more abundant closer to the middle of the CDS for A and U nucleotides. Approximately 9.5% of the putative modification regions contained a previously identified pseudouridine modification motif within ± 4 nt [58]. Most of the modified mRNAs were involved in central functions, such as translation, transcription, central metabolism, and transport (Fig. 2E, Supplementary Fig. S2E), indicating that stress-response genes were not primarily regulated at the epitranscriptomic level during the early stress response.

### rRNAs undergo consistent epitranscriptomic changes across stress conditions

rRNA modification sites have previously been characterized in *E. coli*; however, stress-induced changes in modification abundance remain unclear. Using an IVT sample to control for systemic noise, we analyzed the oxidative-, acid-, and no-stress samples using the proportion of basecalling error (BCError) at each rRNA position to detect modification presence/absence. This approach allowed detection of 92% of the 36 known modified positions in the 23S and 16S (Supplementary Fig. S3A, B). Because BCError can be used as a proxy for modification abundance [19, 39, 51, 59, 60], we calculated normalized changes in rRNA modification levels between stress-treated (BCError_stress_) and no-stress (BCError_no stress_) samples as follows:


\begin{eqnarray*}
\Delta \mathrm{BCError} = {\mathrm{BCError}}_{\mathrm{stress}}- {\mathrm{BCError}}_{\text{no stress}}
\end{eqnarray*}

\begin{eqnarray*}
\mathrm{Normalized}\Delta \mathrm{BCError} = \frac{\Delta \mathrm{BCError}}{{\mathrm{BCError}}_{\text{no stress}}}
\end{eqnarray*}

**Figure 3.**
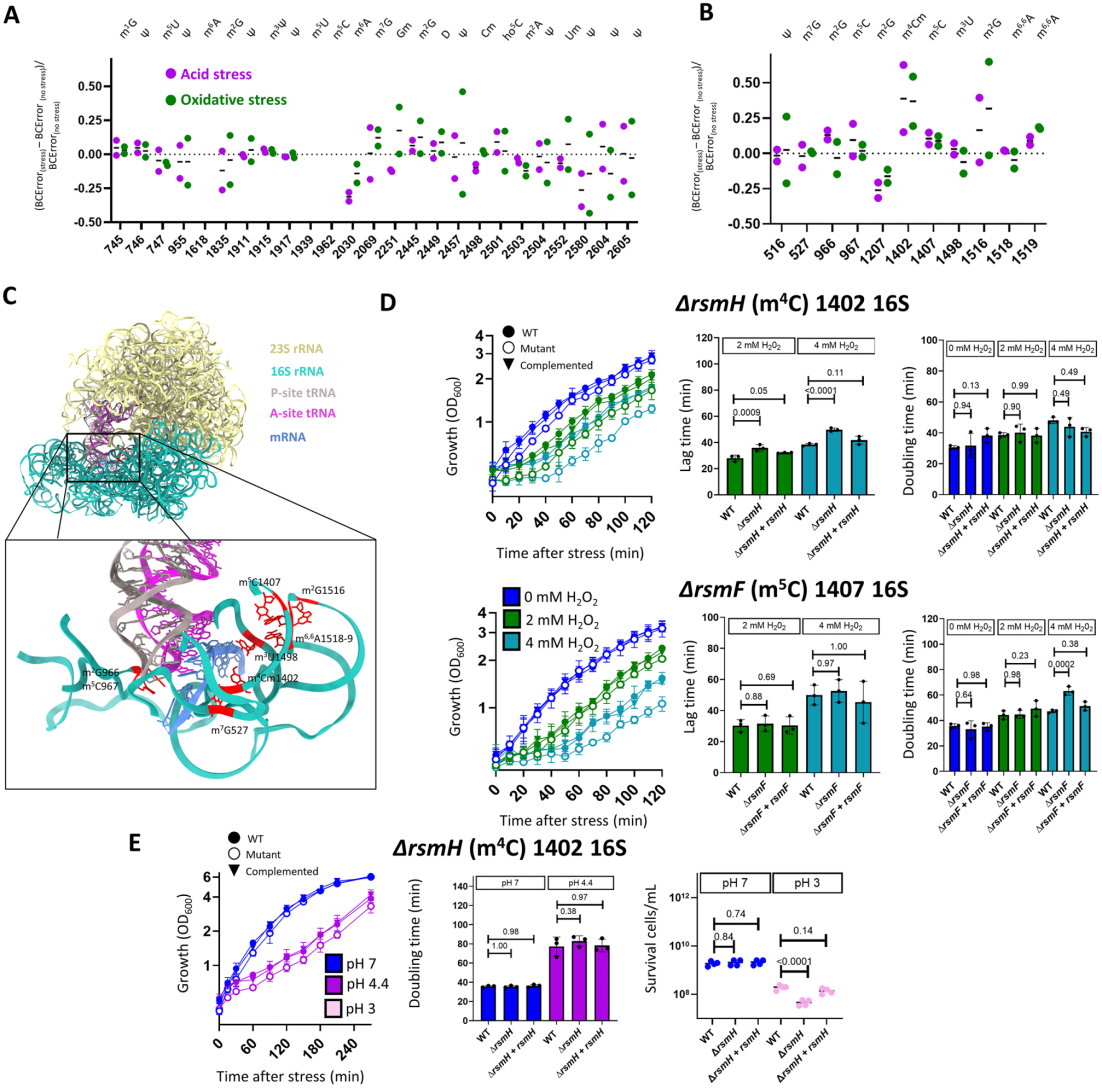
Epitranscriptomic changes in rRNA. (**A, B**) Relative changes in (**A**) 23S and (**B**) 16S rRNA modification levels in acid- and oxidative-stress samples compared to the no-stress control based on nanopore sequencing data. Values for each position were calculated as ΔBCError (BCError in the stress sample minus BCError in the no-stress control) divided by BCError in the no-stress control to normalize the data. Data are shown for known modification sites in the *E. coli* rRNA. (**C**) Schematic showing the P and A sites of the ribosome with RNA modifications around an example mRNA (PDB 7k00). The illustration demonstrates the three-dimensional locations of the modification sites discussed in this study. (**D**) Phenotypic analyses of wild-type (WT) *E. coli* and Δ*rsmH* (m^4^C1402) and Δ*rsmF* (m^5^C1407) mutants under oxidative stress (2 or 4 mM H₂O₂), comprising growth rate (left), lag time (center), and doubling time (right). Lag time and doubling time were calculated from growth rate data. (**E**) Phenotypic analyses of WT and Δ*rsmH* (m^4^C1402) *E. coli* under acid stress (pH 4.4), comprising growth rate (left), doubling time (center), and survival (right). Doubling time was calculated from growth rate data. *P*-values calculated with two-way analysis of variance (ANOVA) and post-hoc Dunnett’s multiple comparisons test. Differences were considered statistically significant at a threshold of *P* ≤ 0.05.

The 16S rRNA showed increased m^4^Cm1402 and m^5^C1407 levels under both stress conditions (Fig. 3B), as confirmed with MS (Supplementary Fig. S3A–H). An m^5^C Rol-LAMP assay was used to experimentally validate these results at the single-nucleotide level. Under acid-stress conditions, the results were consistent with the sequencing data, demonstrating an increase in m^5^C only at position 1407 (Supplementary Fig. S3I). Other changes (e.g. an increase in m^6,6^A at position 1519 of the 16S) were observed with sequencing but not confirmed by MS.

Notably, modifications with stress-induced changes were located near the ribosomal decoding center (Fig. 3C). To assess whether these modifications were required for stress responses, we generated knockout mutants for *rsmI* and *rsmH* (which are responsible for m^4^Cm1402), *rsmF* (m^5^C1407), *rsmJ* (m²G1516), and *rsmA* (m^6,6^A1518–1519), and compared growth phenotypes at 2 and 4 mM H_2_O_2_ (Fig. 3D, Supplementary Fig. S4). Oxidative stress induced a longer lag phase in ∆*rsmH* and slower growth in Δ*rsmF* compared to the WT (Fig. 3D). These results suggested that the corresponding modifications (m^4^C1402 and/or m^5^C1407) may function in the early oxidative stress response in *E. coli*. In contrast, the ∆*rsmI*, ∆*rsmJ*, and ∆*rsmA* knockouts showed no significant differences in growth compared to the WT under oxidative stress (Supplementary Fig. S4A). ∆*rsmH* cells had a significant decrease in bacterial growth (i.e. biomass) and survival compared to the WT under acid stress (Fig. 3E). The ∆*rsmI*, ∆*rsmF*, and ∆*rsmA* knockouts showed no differences under acid stress (Supplementary Fig. S4B–D). Overall, these results indicated that changes in rRNA modification levels were essential for overcoming growth inhibition (lag phase) during the early stress response.

### Changes in the tRNA epitranscriptome are stress-specific

Changes in tRNA modification abundance were assessed using two sample types (Fig. 2A): mRNAe (described above) and Nano-tRNAseq (generated with a tRNA-specific library preparation kit [39]) (Supplementary Fig. S5A). mRNAe samples were expected to contain a mixture of unprocessed, immature, and mature tRNAs, together called pre-tRNA, whereas the Nano-tRNAseq samples should be enriched in mature, aminoacylated (charged) tRNAs. Supporting this hypothesis, most Nano-tRNAseq reads were ∼70–100 nt in length (consistent with the mature tRNA size); mRNAe samples showed a much higher proportion of reads from 100–500 nt, corresponding to unprocessed and immature tRNAs (e.g. those that had not yet been cleaved of the leader and/or trailer sequences or other RNAs within the same operon) [13] (Supplementary Fig. S5B). Comparison of BCError between mRNAe and Nano-tRNAseq samples revealed higher average levels of each modification type in Nano-tRNAseq samples (Supplementary Fig. S5C). Similarly, modification levels at each individual site across the tRNAs were generally higher in Nano-tRNAseq samples (Supplementary Fig. S5D). Although there were no stress-dependent changes in tRNA expression (Supplementary Fig. S5E, F), there were differences in relative tRNA abundance between the pre-tRNA and Nano-tRNAseq samples (Supplementary Fig. S5G), further demonstrating that the two preparation methods captured distinct tRNA subpopulations.

**Figure 4.**
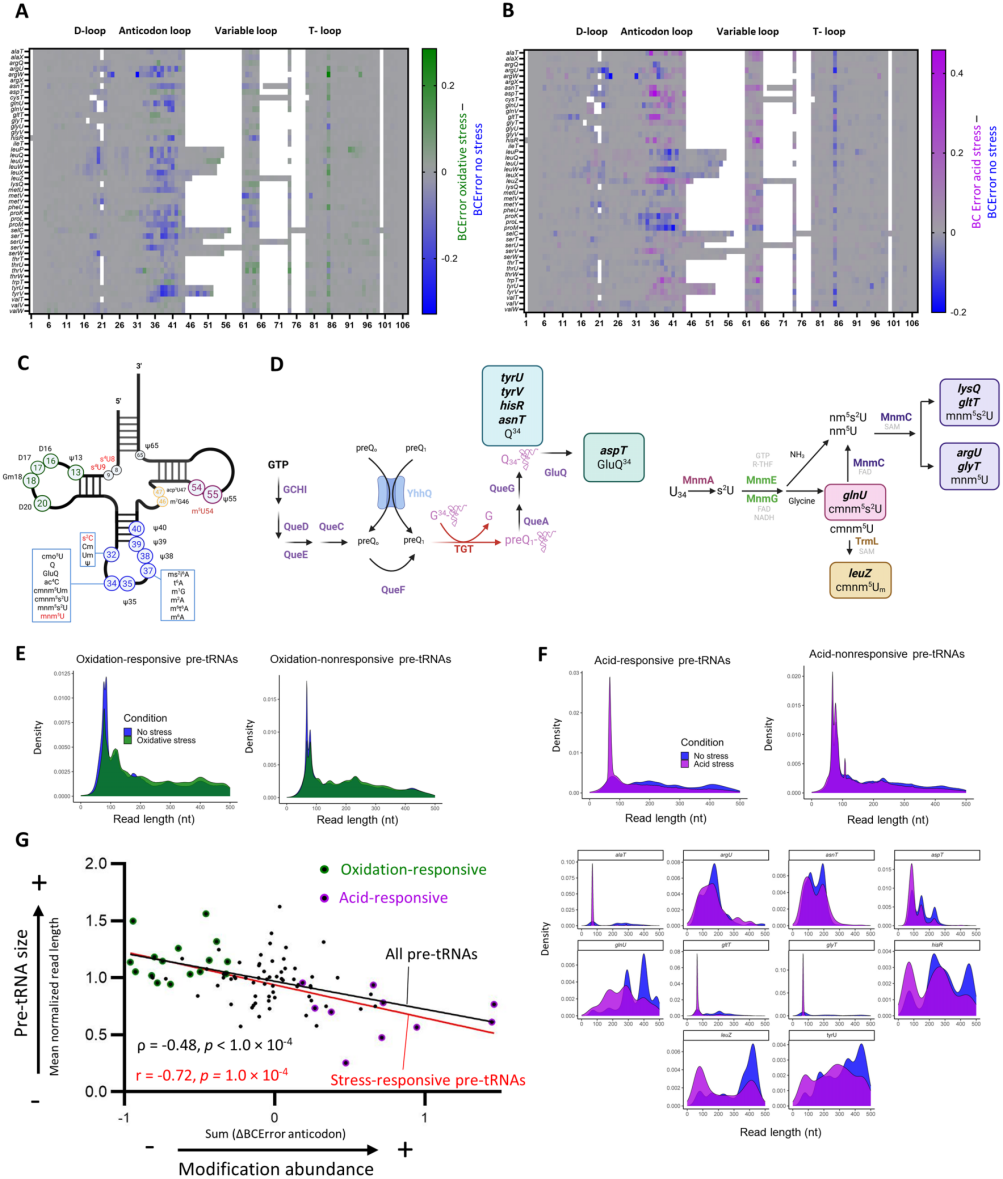
Epitranscriptomic changes in tRNAs. (**A, B**) Changes in uncharged *E. coli* pre-tRNA modification abundance in response to (**A**) oxidative- and (**B**) acid-stress conditions. Changes in uncharged pre-tRNA abundance at each tRNA position were calculated as BCError in the oxidative- or acid-stress sample minus BCError in the no-stress control for all (**A**) 46 and (**B**) 45 unique tRNAs present at a sufficient read depth (≥ 5 reads) and containing modifications that were detected with BCError. Data are shown as the average values from two biological replicates. Positions shown in blue had greater modification abundance under no-stress conditions compared to stress conditions; positions shown in green (**A**) or purple (**B**) had greater modification abundance under the indicated stress condition. (**C**) Two-dimensional rendering of a model tRNA, showing the types and positions of characterized modifications in *E. coli* tRNAs. Created in BioRender. https://BioRender.com/c46l040. (**D**) Schematic representations of the biosynthetic pathways from GTP to queuosine (Q) (left) and UTP to Mnm (right). Q and Mnm are incorporated into five and six tRNAs, respectively. Nine pre-tRNAs identified in the present study as having increased modification abundance in the wobble position under stress conditions are modified via either the Q or Mnm pathway. Created in BioRender. https://BioRender.com/z98jjq7. (**E, F**) Normalized pre-tRNA abundance at read lengths from 0–500 nt. Distributions are shown for (**E**) all pre-tRNA species under no-stress and oxidative-stress conditions, separated by oxidative-stress responsiveness, and (**F**) all pre-tRNA species under no-stress and acid-stress conditions, separated by acid-stress responsiveness (upper) and all acid-stress-responsive pre-tRNAs individually (lower). Each pre-tRNA, biological replicate, and sample condition was weighted equally. Longer read lengths generally indicate that a pre-tRNA is in an earlier stage of processing, i.e. that it has not yet been fully cleaved. (**G**) Relationships between pre-tRNA read length and anticodon loop modification abundance. Among stress-responsive tRNAs, there was an inverse correlation between read length and anticodon loop modification abundance, indicating that longer pre-tRNA reads were in a relatively early stage of tRNA maturation. The values for all pre-tRNAs were non-normally distributed, whereas the values for the stress-responsive pre-tRNAs alone were normally distributed (as determined with a Shapiro–Wilk test). The strengths of the relationships for all pre-tRNAs (black) and stress-responsive pre-tRNAs only (red) were therefore assessed with Spearman’s and Pearson correlations, respectively. Correlations were considered statistically significant at a threshold of *P* ≤ 0.05.

To evaluate whether stress caused changes in tRNA modification levels, we compared BCError by calculating the difference between stressed samples and no-stress controls as previously described [39, 51, 61]. Stress exposure changed tRNA modification patterns only in the pre-tRNAs from mRNAe samples (Fig. 4A, B; Supplementary Fig. S6A–D), not in the charged tRNAs isolated with Nano-tRNAseq (Supplementary Fig. S7A, B). Furthermore, MS was performed on a purified fraction of RNAs (≤200 nt) containing primarily mature tRNAs (comparable to those isolated with Nano-tRNAseq) to assess the general abundance of selected modifications. Very few differences in tRNA modification levels under oxidative and acid stress were found with MS (Supplementary Fig. S8A–P), consistent with the Nano-tRNAseq sequencing data.

In mRNAe samples, oxidative stress led to a pronounced decrease in pre-tRNA modifications within the anticodon loop of 16 tRNAs (*argU, argW, asnT, glnU, leuQ, leuU, leuW, metU, proK, proL, proM, selC, serT, serV, tyrU*, and *tyrV*) (Fig. 4A), which were thus considered oxidative-stress responsive. There were no significant differences in tRNA expression between control and stress conditions (Supplementary Fig. S5E, F), indicating that stress-dependent changes were solely at the epitranscriptomic rather than the transcriptomic level. Acid stress produced a distinct response, with 10 pre-tRNAs exhibiting increased modifications at the wobble position (nucleotide 34) (Fig. 4B, C). Notably, nearly all of these 10 acid-responsive tRNAs have wobble-position modifications that are generated via the queuosine (Q) pathway [62] (*aspT, asnT, hisR*, and *tyrU*) (Fig. 4D, Supplementary Fig. S9A) or the Mnm pathway [63, 64] (*argU, glnU, glyT, gltT*, and *leuZ*) (Fig. 4D, Supplementary Fig. S9B). *alaT* was the only acid-responsive tRNA that was not modified via the Q or Mnm pathway, but instead via the 5-methoxycarbonylmethoxyuridine (cmo^5^U) pathway [65].

We then explored changes in tRNAs between treatments in the mRNAe preparations. In each treatment group, a majority of the sequenced tRNAs were very short (<100 nt), corresponding to highly processed, mature tRNAs; however, populations of longer transcripts (corresponding to immature tRNAs) were also present. To assess how stress conditions affect the abundance of mature and immature tRNAs, we compared the tRNA read length distributions among stress-responsive and stress-nonresponsive tRNAs as classified above (Fig. 4E, F). Surprisingly, the oxidative-stress-responsive pre-tRNAs were enriched in less processed molecules under oxidative-stress conditions compared to no-stress conditions, whereas stress-nonresponsive pre-tRNAs showed similar distributions under both oxidative-stress and no-stress conditions (Fig. 4E). In contrast, the acid-stress-responsive tRNAs showed an accumulation of mature molecules under acid stress (Fig. 4F). Thus, oxidative stress led to decreased levels of specific anticodon modifications and a decreased abundance of processed pre-tRNAs overall. In contrast, acid stress was associated with increased abundance of processed pre-tRNAs bearing acid-responsive anticodon modifications.

These findings suggest that altered anticodon modification levels are associated with stress-induced changes in pre-tRNA maturation. Using the average length of each pre-tRNA under acid-stress conditions compared to no-stress conditions as a proxy for pre-tRNA maturity and the total ΔBCError for the anticodon as a comprehensive measure of modification levels, we tested for a correlation between anticodon modification abundance and pre-tRNA maturity. There was a moderate correlation (ρ = −0.48, *P *< 1.0 × 10^−4^) among all pre-tRNAs, which increased to a strong correlation (r = −0.72, *P *= 1.0 × 10^−4^) when only stress-responsive pre-tRNAs were considered (Fig. 4G). These data supported our hypothesis of stress-induced regulation of pre-tRNA maturation during the early stress response.

### Wobble-position modifications regulate translation of a subset of proteins under stress

The decoding process uses both Watson–Crick and non-Watson–Crick interactions for codon–anticodon pairing; the former does not require modifications in the anticodon for correct codon–anticodon interactions, whereas the latter are anticodon modification dependent. The observed increase in wobble-position modifications via the Q and Mnm pathways may indicate the presence of a stress-resistance mechanism via differential abundance of modification-dependent and -independent codons. To evaluate the effects of anticodon modifications on acid stress survival, we assessed the relationship between translational efficiency (TE) under acid stress [5] and modification-dependent codon abundance. Notably, transcripts with increased TE at pH 4.4 versus 7.6 had a higher abundance of modification-dependent codons than transcripts with decreased TE (Supplementary Fig. S9C). This effect was not observed at pH 5.8 (Supplementary Fig. S9D), indicating that the mechanism was only in operation under severe acid stress.

**Figure 5.**
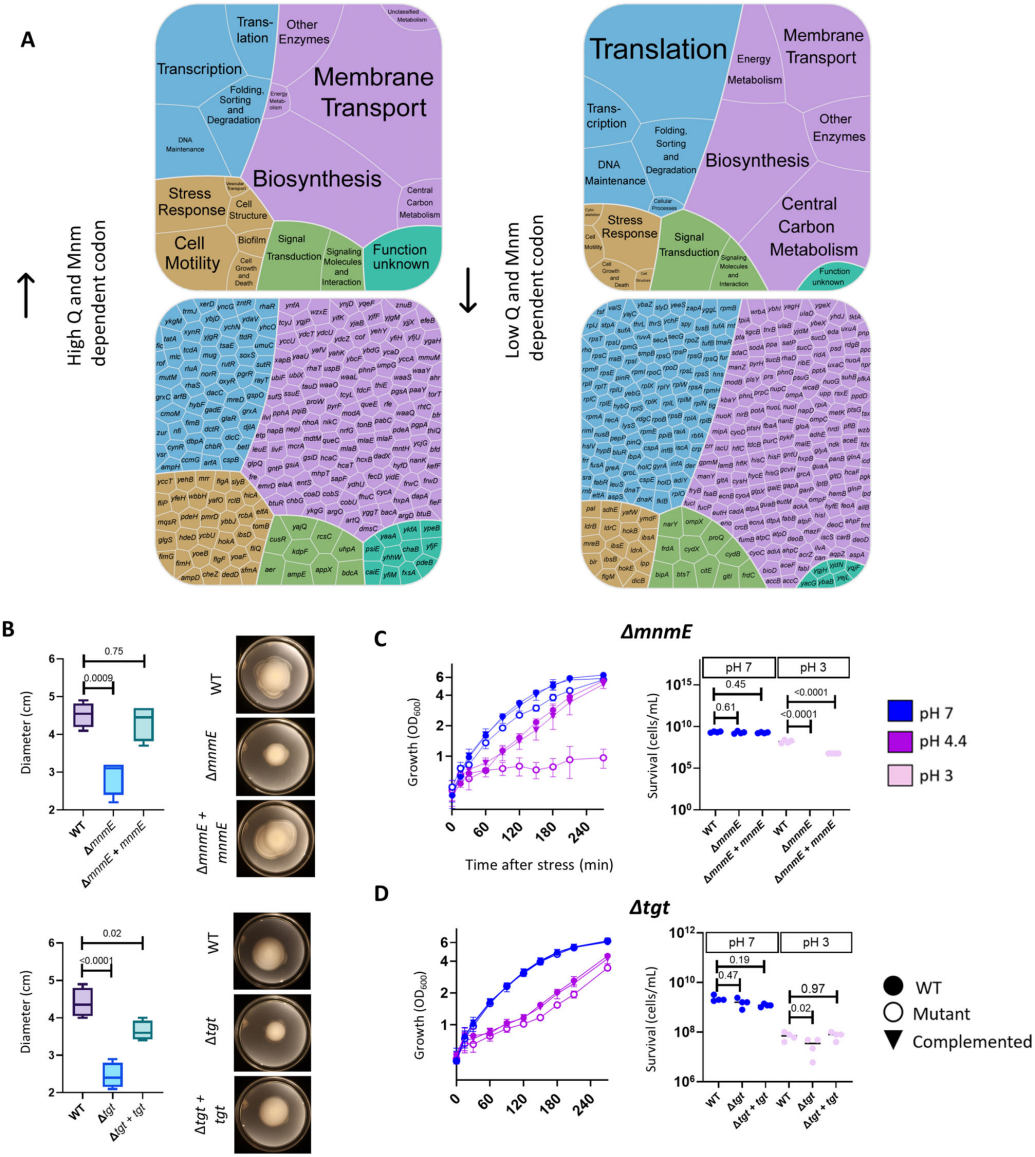
Modifications in the wobble position are implicated in *E. coli* stress recovery. (**A**) Functional annotations of the ∼400 transcripts with the highest (left) and lowest (right) relative abundance of Q- and Mnm-dependent codons. Cell color corresponds to the highest-level annotation for each gene as defined in KEGG: Genetic Information Processing (blue), Metabolism (purple), Cell Processes (gold), Environmental Information Processing (green), or Function unknown (teal). Genes having the KEGG annotation “Function unknown” were annotated based on a literature search where possible. (**B**) Swimming motility of Δ*mnmE* (Mnm pathway) and Δ*tgt* (Q pathway) mutants compared to the WT. Swimming motility was quantified as colony diameter. Images at right depict one representative sample each. Motility data are shown as the average of four biological replicates. *P*-values calculated with one-way ANOVA and post-hoc Dunnett’s multiple comparisons test. Differences were considered statistically significant at a threshold of *P* ≤ 0.05. (**C, D**) Left, growth of (**C**) Δ*mnmE* and (**D**) Δ*tgt* mutants under acid stress (pH 4.4) compared to no-stress conditions. OD_600_ was used as a measure of bacterial growth. Right, cell survival after acid shock assays. Differences between strains were assessed using two-way ANOVA with post-hoc Dunnett’s multiple comparisons test and considered statistically significant at a threshold of *P* ≤ 0.05.

We next ranked all *E. coli* transcripts by the relative abundance of modification-dependent codons (see Materials and methods) to determine whether MoTTs regulation may occur under acid stress. The 400 transcripts with the highest abundance of Q- and Mnm-pathway modification-dependent codons (Supplementary Table S6) were enriched in functions associated with stress-related responses; the transcripts included two encoding membrane stress proteins of the phospholipid trafficking system (*mlaB* and *mlaF*) [66]; efflux pumps (*emrB, embD*, and *entS);* lipopolysaccharide biosynthesis genes (*waaO, waaQ*, and *waaY*); transcription factors, mainly those related to stress responses (e.g. oxidative or reactive nitrogen species responses, such as *oxyR, soxS*, and *norR*); and mobility (*flgC, flgF, flgA, fliP, fliQ*, and *cheZ*) (Fig. 5A). Conversely, the ∼400 transcripts with the lowest abundance of Q- and Mnm-pathway modification-dependent codons (Supplementary Table S6) were generally related to constitutively expressed *E. coli* genes, including those involved with central carbon metabolism (*pfkA, fbaA, glpX, lpd*, and *gapA*); translation (particularly 30S ribosomal subunit proteins, namely *rpsU, rpsA, rpsL, rpsC, rpsB, rpsI, rpsQ, rpsT, rpsE, rpsG, rpsP, rpsO*, and *rpsS*, and 50S ribosomal subunit proteins, namely *rplL, rplM, rplW, rplA, rplI, rplY, rplC, rplX, rplJ, rplT, rplO, rplK, rplB, rplE, rplS, rplN*, and *rplU)*; and transcription factors related to the RNA polymerase component (*rpoB, rpoC, rpoD*, and *rpoZ*) (Fig. 5A). Thus, although both datasets contained approximately equal numbers of transcripts with the primary annotation “Stress Response” (Fig. 5A), transcripts with a high abundance of Q- and Mnm-dependent codons tended to be known stress-responsive genes, whereas transcripts with a low abundance of modification-dependent codons tended to be involved in basal cellular functions. This enrichment of stress-responsive functions among transcripts with a high abundance of modification-dependent codons supported the idea that MoTTs regulation is involved in the early stress response.

One of the most specific functional annotations among transcripts with a high abundance of Q- and Mnm-pathway modification-dependent transcripts was cell motility. To investigate whether tRNA modifications enabling translation of these transcripts were necessary for motility, we phenotypically characterized mutants lacking enzymes required for wobble-position modifications via the Q and Mnm pathways. Specifically, we deleted genes encoding Tgt, an RNA-guanine transglycosylase responsible for Q modification [67]; MnmE, which is involved in mnm^5^s²U biosynthesis [68]; and MnmA, which catalyzes 2-thiouridine formation [28] (Fig. 4D). These mutants showed impaired swimming capacity compared to the WT (Fig. 5B, Supplementary Fig. S9E), demonstrating the essentiality of tRNA modifications in cell motility. Furthermore, ∆*mnmE* mutants displayed a strong growth defect and decreased survival compared to the WT under acid stress (Fig. 5C). The ∆*mnmA* mutant showed a similar phenotype (Supplementary Fig. S9E, F), and although the ∆*tgt* mutant displayed only a minor growth defect at pH 4.4, its survival was impaired at pH 3 (Fig. 5D). These findings confirmed the critical roles of the Q and Mnm pathways [63] in the *E. coli* acid stress response.

## Discussion

Bacteria survive harsh conditions by detecting environmental changes, then employing diverse adaptation strategies. These adaptations involve transcriptional, translational, and post-translational regulation to alter gene expression and cellular metabolism, often mediated by sophisticated regulatory networks, global regulators, and small RNAs [69]. However, there has yet to be a comprehensive, systematic assessment of multiple stress responses at the epitranscriptomic level. To fill this gap, we used DRS to evaluate the epitranscriptomic profile during the early stress response, in which multi-level responses are initiated for survival and adaptation. The use of two abiotic stress conditions, acid and oxidative stress, allowed us to identify components of the early stress response. Overall, we observed both general and stress-specific changes in modification patterns across all RNA biotypes, which were consistently involved in redirection or adaptation of the general functions of *E. coli* (e.g. Fig. 2F). Changes in rRNAs and tRNAs primarily occurred via addition of modifications to pre-synthesized RNAs rather than to newly synthesized molecules. At the mRNA level, there is an increase in stress-related gene expression along with epitranscriptomic changes, suggesting an interplay between the adjustment of general *E. coli* functionality and the specific stress response. These results indicate that epitranscriptomic changes may help bacteria to protect themselves and redirect central functions (and all associated biomolecules) as a first stress response (Fig. 1D). For detection of putative novel modifications in the mRNA and ncRNA, we employed two approaches with distinct mechanisms: one involving evaluation of the BCError associated with each RNA position [51], and one (nanoSundial) that uses raw electrical signal features from nanopore sequencing [50]. There was a high degree of variability between biological replicates, potentially explainable as off-target catalysis by rRNA- and tRNA-modification enzymes due to changes in substrate availability (a well-documented phenomenon in bacteria [19, 70, 71]; Supplementary Fig. S10A). Overall, mRNA modifications were most abundant in the CDS; across all conditions, modifications were primarily found in transcripts associated with central functions (such as carbon metabolism and transcription). Additionally, some known stress-responsive transcripts were found to contain modifications after exposure to acid or oxidative stress. However, due to the relatively low abundance of such transcripts among all putative modification sites, most stress-response genes do not appear to be regulated primarily at the epitranscriptomic level. Recently, some mRNA modifications were found to alter gene expression [58, 70], and a separate study showed that the presence of pseudouridine in mRNAs stabilizes transcripts [58]. Taken together, these findings indicate important functional roles of bacterial mRNA modifications. Our set of putative novel modification sites offers an extensive pool of targets for future validation of such functions.

The rRNA epitranscriptome showed stress-type-independent changes during the early stress response, which were phenotypically detectable as differences in the lag phase and growth rate. The increase we observed in m^5^C1407 of the 16S rRNA is consistent with previous findings that modification levels at this position increase under oxidative stress [37]. Furthermore, the changes observed in both m^4^Cm1402 and m^5^C1407 have also been detected under heat stress [19, 37], demonstrating that these modifications have roles in the early response to multiple stressors. Heat stress leads to an increase in m^6,6^A1518–19 levels [19], but this effect was not observed here under oxidative or acid stress.

Notably, analysis of rRNAs via DRS, MS, and m^5^C Rol-LAMP under acid and oxidative stress revealed stress-dependent changes in modifications only within the rRNA decoding center, consistent with results under heat stress [19]. A recent study demonstrated that m^5^C1407 increases the duration of the excited and ground states, implying that this modification increases the available time for correct codon–anticodon recognition during translational elongation [72]. Thus, rRNA modifications in the decoding center may represent a dynamic control of the decoding process. This hypothesis is supported by our phenotypic mutant analysis, showing that RsmH and RsmF (which catalyze stress-responsive rRNA modifications) are important for both the oxidative- and acid-stress responses. In contrast, levels of other modifications in the decoding center did not change under stress; single mutants lacking the enzymes that produce these modifications (∆*rsmA* and ∆*rsmJ*) showed no abnormal growth phenotype under stress, strongly validating our sequencing results and overall experimental strategy.

These findings emphasized that the epitranscriptomic response is specific for certain modifications present in the *E. coli* decoding center. We therefore propose that increased levels of selected modifications in this region maintain translation fidelity (Supplementary Fig. S10B). Increased modification levels in the decoding center have recently been identified under multiple stress conditions [19, 37], but the source of these changes and the associated mechanisms of stress sensing are not clear. For example, oxidative stress is known to increase *rsmF* transcription [73], potentially leading to higher rRNA modification levels due to increased enzyme abundance. Alternately, there could be an increase in co-transcriptional or post-transcriptional addition of modifications to the rRNAs, unrelated to enzyme abundance. Another explanation is that rRNAs lacking these modifications are less stable and therefore more quickly degraded, resulting in a relative increase in rRNAs bearing decoding center modifications.

To thoroughly assess tRNA modifications during the early stress response, we employed two distinct sample preparation methods. Multiple protocols have been developed specifically for tRNA sequencing, typically involving size-based fractionation, deacylation, and adapter ligation [39, 51, 61] (e.g. Nano-tRNAseq). Alternative protocols rely on general polyadenylation (e.g. our mRNAe sample preparation method), which captures pre-tRNAs together with other RNAs. The first method theoretically yields a higher read number, whereas the second allows for simultaneous sequencing of multiple RNA types within a single sample. Furthermore, the deacylation step required by approaches such as Nano-tRNAseq ensures that the captured tRNA population is enriched in fully mature (charged) tRNAs, in contrast to the mixed populations of immature, partially processed, and mature but uncharged pre-tRNAs obtained with general polyadenylation. Importantly, our work is the first to report tRNA sequencing results from parallel implementation of polyadenylation [19, 50] and deacylation/primer ligation [39, 51] protocols, capturing tRNA and pre-tRNA.

Detected tRNA modification levels varied considerably between Nano-tRNAseq and mRNAe samples. The higher number and abundance of modifications detected in Nano-tRNAseq samples were consistent with previously published data indicating that pre-tRNAs undergo sequential processing and modification to reach a form that is available for aminoacylation [13]. However, the mature, charged tRNAs captured with Nano-tRNAseq showed no statistically significant differences in modification levels under stress conditions compared to no-stress controls. In contrast, pre-tRNAs from the mRNAe preparation showed a huge decrease in anticodon modifications under oxidative stress, consistent with effects previously observed under heat stress [19]. *E. coli* tRNA levels are greatly decreased at 5 min after H_2_O_2_ exposure, diminishing translation elongation [4]. Therefore, the pre-tRNAs present in mRNAe samples were presumably not processed. Supporting this idea, oxidative-stress samples showed enrichment of longer tRNA transcripts compared to no-stress samples. This suggests an abundance of relatively early-stage, immature pre-tRNAs with low anticodon modification levels at 15 min after oxidative stress (Supplementary Fig. S10C), and the decreases in translation elongation can therefore likely be explained by both the overall lower tRNA abundance and reduced modification levels.

Compared to oxidative stress, a distinct pattern was observed in pre-tRNAs under acid stress; although there was a decrease in pseudouridine levels in the T-loop, some sites in the anticodon wobble position showed increased modification levels. These acid-responsive pre-tRNAs, primarily regulated by the Q and Mnm pathways, were enriched in processed (i.e. relatively short and highly modified) pre-tRNAs, which were available for aminoacylation. Generally, transcripts with a higher TE under acid stress had a greater abundance of codons requiring acid-responsive anticodon modifications for translation compared to transcripts with decreased TE, implicating stress-induced MoTTs regulation. The data suggest that high proportions of processed pre-tRNAs may be required to support a high tRNA demand. A previous comparative proteomic analysis of a knockout mutant for *queF*, a gene in the Q biosynthetic pathway, showed decreased levels of GadE, the main transcriptional regulator of the GAD system, in addition to other acid-stress-related proteins, including MdtE, MdtF, HdeD, GadA, and GadB [74]. This finding supports the involvement of Q in the acid-stress response.

Our study provides the first comprehensive description of a multifaceted epitranscriptomic response in *E. coli* during the early response to two stress conditions. We uncover simultaneous epitranscriptomic changes in the mRNA, pre-tRNA, tRNA, and rRNA, revealing a spectrum of regulatory events that range from stress-type-independent signatures to stress-specific adaptations. These findings highlight epitranscriptomic regulation as a previously underappreciated layer of the bacterial stress response, offering new insights into how bacteria rapidly reprogram their physiology to survive environmental challenges. By generating the most comprehensive dataset of RNA modifications in a model prokaryote to date, this work establishes a foundation for understanding the presence, abundance, and functional impacts of epitranscriptomic changes during the earliest stage of stress adaptations.

## Supplementary Material

gkag042_Supplemental_Files

## Data Availability

Sequencing data have been deposited at NCBI Gene Expression Omnibus (GEO) under accession number GSE309032. This study does not describe any novel programs, software, or algorithms.
