# Supplementary material for: Epitranscriptomic control of stress adaptations in Escherichia coli
Source: Nucleic Acids Res. 2026 Feb 2;54(3):gkag042. doi: 10.1093/nar/gkag042 (PMC12862378; doi:10.1093/nar/gkag042)
Supplement: gkag042_Supplemental_Files [file gkag042_supplemental_files.zip › Supplementary data with link fig 30012026.pdf]

Epitranscriptomic control of stress adaptations in *Escherichia coli*

Sebastián Riquelme-Barrios[1], Siobhan A. Cusack[1], Luis Rivera-Montero[1], Leonardo Vásquez-Camus[1], Korinna Burdack[1], Sophie Brameyer[1], Maximilian Berg[2], G. Nur Yeşiltaç-Tosun[2], Stefanie Kaiser[2], Pascal Giehr[3], Kirsten Jung[1]*

[1]Faculty of Biology, Microbiology, Ludwig-Maximilians-Universität München, 82152 Martinsried, Germany

[2]Goethe University Frankfurt, Faculty 14, Institute of Pharmaceutical Chemistry, 60438 Frankfurt, Germany

[3]Department of Chemistry, Ludwig-Maximilians-Universität München, 81377 München, Germany

*To whom correspondence should be addressed:

Prof. Dr. Kirsten Jung

Tel: + 49 89/2180-74500 21 Fax: + 49 89/2180-74520

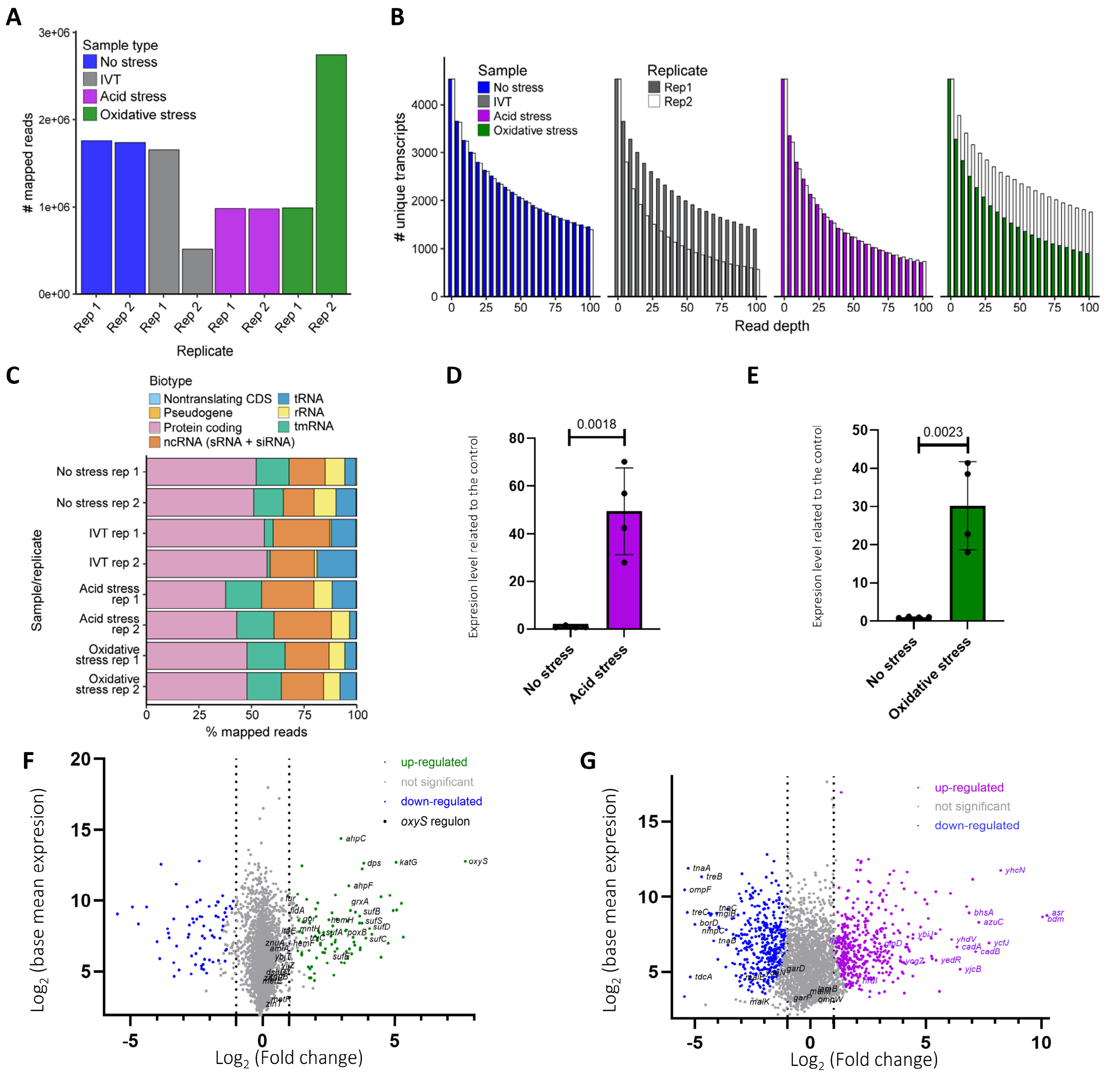

Supplementary Figure 1. Sequencing quality analyses and differential gene expression in DRS data. (**A**) Number of mapped reads produced from ONT sequencing of *E. coli* exposed to no stress, acid stress, or oxidative stress. Data are also shown for an *in vitro*-transcribed (IVT) control sample produced from the no-stress sample. (**B**) Number of unique transcripts detected in the no-stress, IVT, acid-stress, and oxidative-stress samples across a range of minimum read depth thresholds. (**C**) Relative abundance of reads mapped to distinct RNA biotypes in each sample. tRNA, transfer RNA; rRNA, ribosomal RNA; tmRNA, transfer-messenger RNA; ncRNA, noncoding RNA; sRNA, small RNA; siRNA, small interfering RNA. (**D, E**) Expression of (**D**) *cadB*, which encodes a lysine/cadaverine antiporter, under acid stress and (**E**) *katG*, which encodes catalase/hydroperoxidase, under oxidative stress compared to the no-stress control. Expression was measured with quantitative reverse transcription quantitative PCR (qRT-qPCR) and normalized to the 16S rRNA as an internal control. Significant differences were assessed with Student's *t*-test. (**F, G**) Differentially expressed genes (DEGs) in (**F**) oxidative- and (**G**) acid-stressed *E. coli* compared to a no-stress control. Data are displayed as log-

transformed base mean expression vs. $\log_2$(fold change). In (**F**) DEGs controlled by the *oxyS* regulon are labeled; in (**G**) the 20 most strongly up- and down-regulated genes under acid stress conditions as previously described (5) are indicated for comparison.

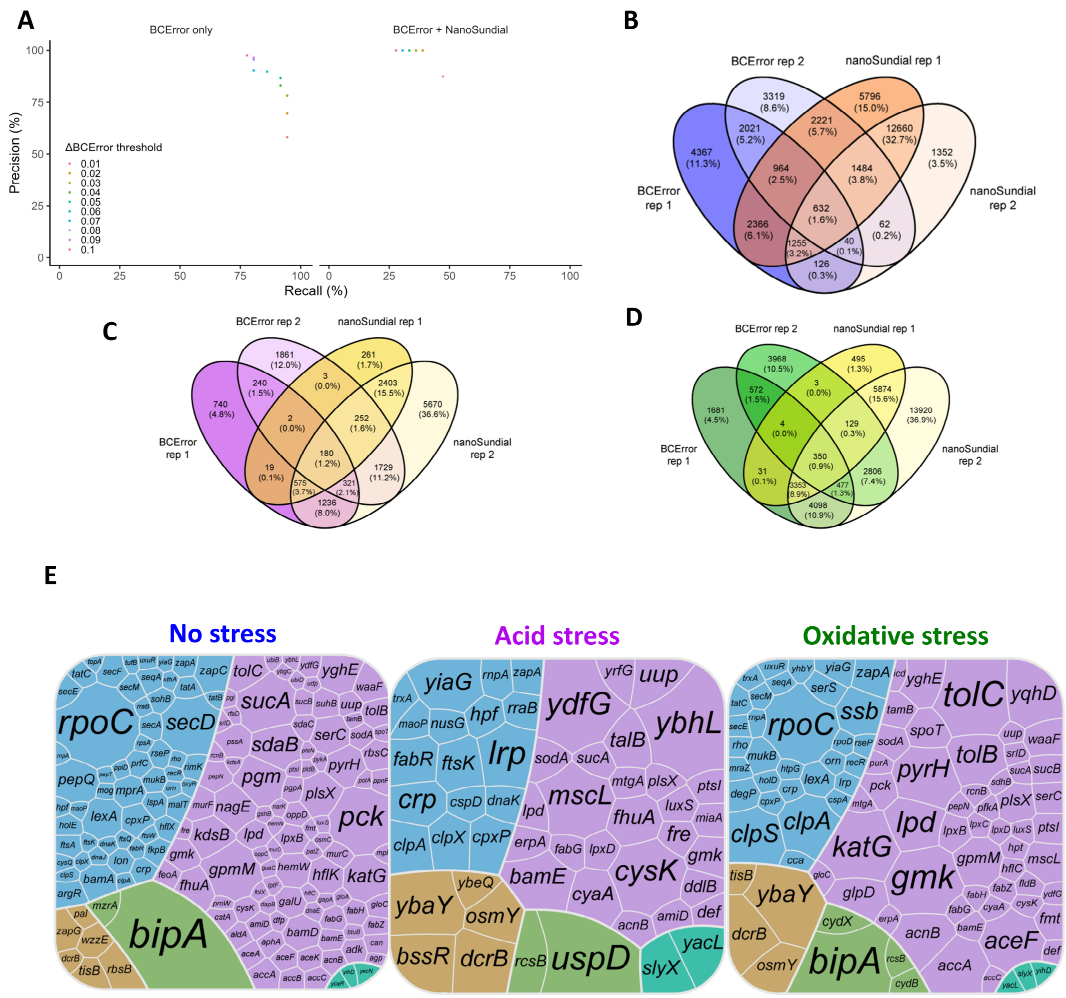

Supplementary Figure 2. Detection of putative novel mRNA and ncRNA modifications in *E. coli* via DRS. (**A**) Performance achieved using (left) BCError alone and (right) BCError plus nanoSundial to detect known *E. coli* rRNA modifications. (**B–D**) Number of putative modification sites identified in the (**B**) no-stress, (**C**) acid-stress, and (**D**) oxidative-stress samples. Putative modification sites/regions were identified in two biological replicates of each sample type using two modification detection approaches, BCError and nanoSundial. The putative modification sites/regions were considered in further analyses only if they were present in both biological replicates and detected with both approaches. (**E**) Identities of transcripts containing modifications. Cell color corresponds to the highest-level annotation for each gene as defined in the Kyoto Encyclopedia of Genes and Genomes (KEGG): Genetic Information Processing (blue), Metabolism (purple), Cell Processes (gold), Environmental Information Processing (green), or Function unknown (teal). Cell size corresponds to the number of putative modified sites or regions in the transcript.

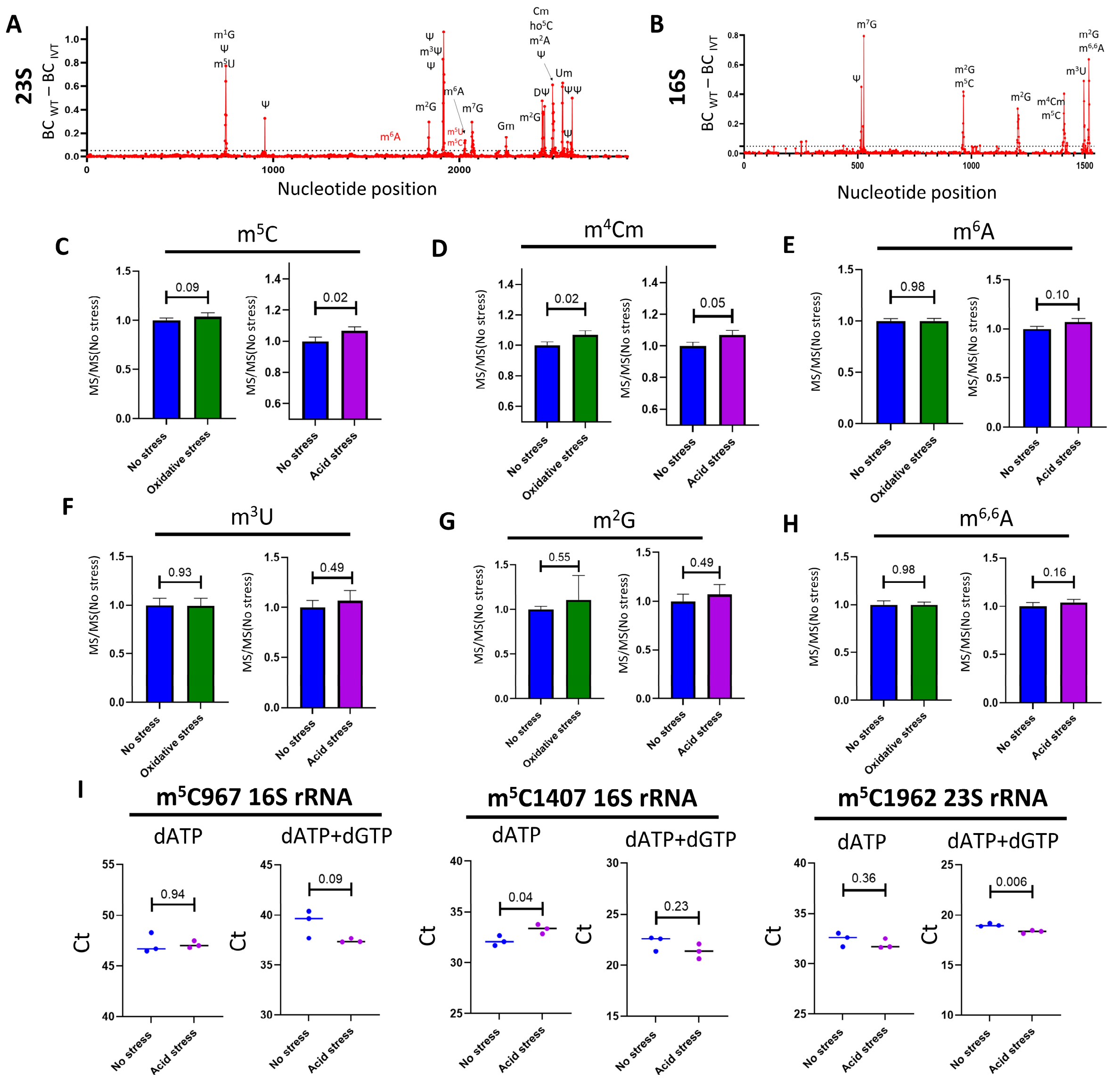

Supplementary Figure 3. Orthogonal validation of changes in rRNA modifications. Detection of rRNA modifications in the (**A**) 23S and (**B**) 16S rRNA of *E. coli.* Values were calculated as the total percentage of basecalling error (BCError) at each site in no-stress samples minus the corresponding BCError value for each site in the IVT control. (**C–H**), MS-based quantification of selected modification types in rRNA samples. Values were calculated as modification abundance in each sample divided by abundance in the no-stress control ($MS/MS_{No\ stress}$) in four biological replicate samples. Error bars indicate the standard deviation of the mean. Significant differences were assessed with ratio paired Student's *t*-test and considered statistically significant at a threshold of $p < 0.05$. (**I**) Quantification of changes in m^{5}C modification levels under acid stress for every reported m^{5}C site in *E. coli* rRNA via m^{5}C Rol-LAMP. Total RNA was analyzed using the indicated site-specific padlock probes with dATP and dATP+dGTP in the ligation step. Data are shown for three biological replicates per sample type. Differences were evaluated with unpaired Student's *t*-test and considered statistically significant at a threshold of $p \leq 0.05$.

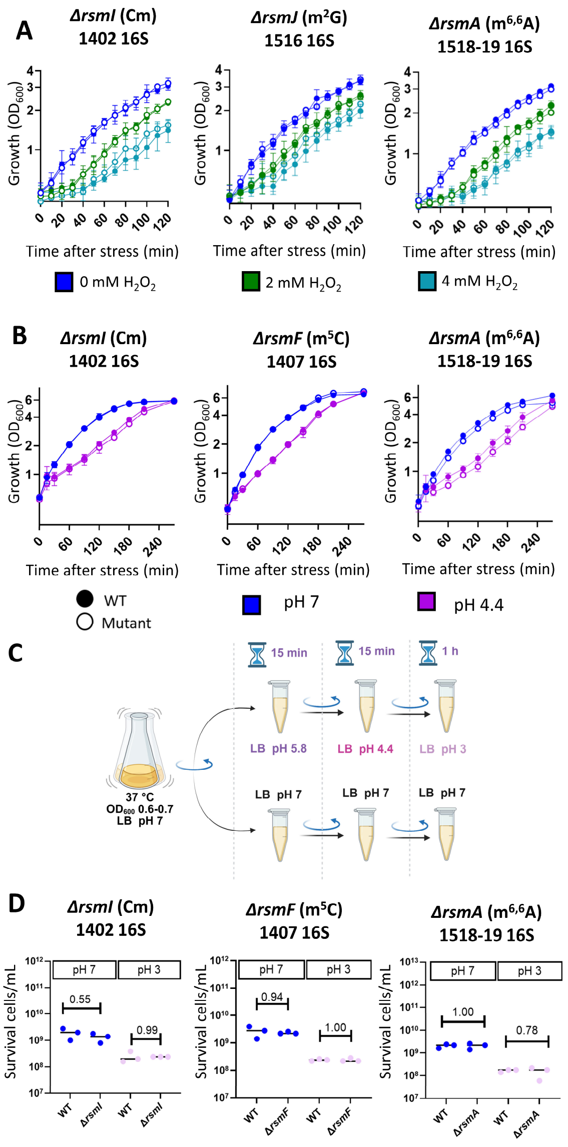

Supplementary Figure 4. Phenotypic characterization of rRNA modification enzyme knockout mutants. (**A**) Phenotypic analyses of WT *E. coli* and mutants that are unable to form known rRNA modifications, namely Δ*rsmI* (Cm1402 in the 16 rRNA), Δ*rsmJ* (m^2G1516 in the 16S rRNA), and Δ*rsmA* ($m^{6,6}$A1518–19 in the 16S rRNA). All strains were exposed to oxidative stress (2 or 4 mM H_2O_2). (**B**) Phenotypic analysis of WT *E. coli* and the rRNA modification enzyme mutants Δ*rsmI* (Cm1402 in the 16 rRNA), Δ*rsmF* (m^5C1407 in the 16S rRNA), and Δ*rsmA* ($m^{6,6}$A1518–19 in the 16S rRNA) under acid stress. (**C**) Scheme for acid shock assays (pH 3); briefly, cultures were grown at pH 7 or treated with a stepwise acid stress protocol (15 min at pH 5.8 followed by 15 min at pH 4.4) prior to exposure to LB medium at pH 3 for 1 h. Cultures were then serially diluted and plated on LB agar. Created in BioRender. https://BioRender.com/68s19x7. (**D**) Survival of WT, Δ*rsmI*, Δ*rsmF*, and Δ*rsmA* cells after acid shock treatment. *p*-values were calculated with two-way ANOVA followed by Šidák correction for multiple comparisons. Differences were considered statistically significant at a threshold of $p \leq 0.05$.

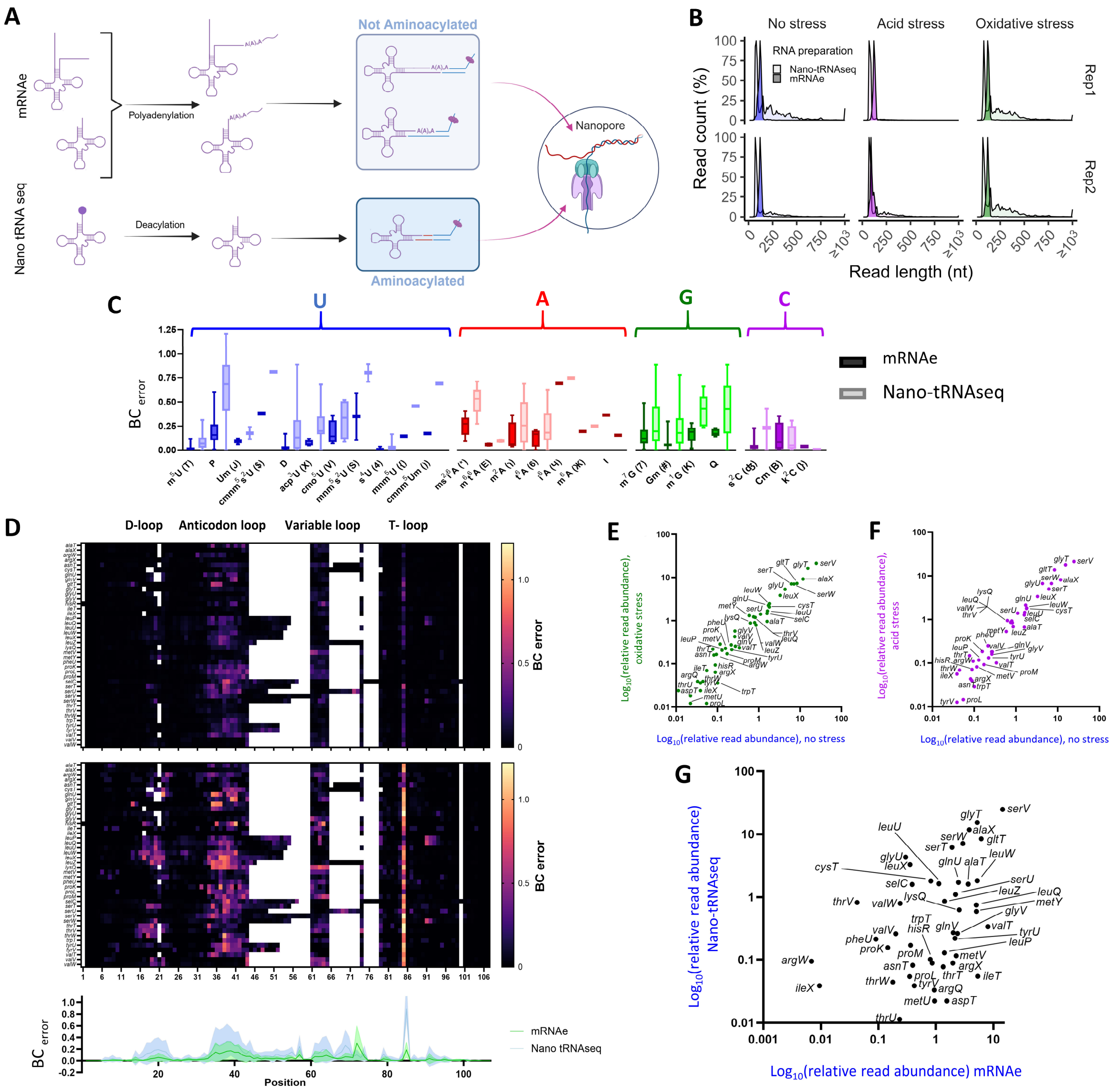

Supplementary Figure 5. Nanopore DRS revealed distinct tRNA subpopulations. (**A**) Graphical representation of the two protocols used for tRNA sequencing via nanopore. The mRNA-enriched (mRNAe) protocol captures uncharged pre-tRNAs. This includes fully immature (unprocessed) pre-tRNAs, which retain untranslated regions (UTRs) and/or lack the modifications required for aminoacylation; tRNAs at all stages of the maturation process; and fully processed, mature but uncharged pre-tRNAs. In contrast, Nano-tRNAseq selects for aminoacylated tRNAs before library preparation, yielding samples that are highly enriched in mature tRNAs. Created in BioRender. https://BioRender.com/6s0vmvc. (**B**) Read length distribution for sequences mapping to the tRNA in two biological replicates of each sample type. Samples prepared with the Nano-tRNAseq protocol produced primarily reads of around 100 nt in length. Samples prepared with the mRNAe protocol produced the largest volume of pre-tRNA reads around 90 nt, with a lower, broader peak from 90–500 nt. (**C**) Comparison of BCError at sites with known modifications in no-stress samples prepared with the mRNAe and Nano-tRNAseq protocols. (**D**) BCError at every nucleotide position of tRNAs detected

in each sample type prepared with the mRNAe (upper) and Nano-tRNAseq (lower) protocols. Biological replicate samples were combined and a minimum depth of five reads was applied for inclusion in the analysis. (**E, F**) Average relative abundance of reads mapped to each unique tRNA under (**E**) oxidative- or (**F**) acid-stress conditions compared to the no-stress control. (**G**) Average relative abundance of reads mapped to each unique tRNA in no-stress samples prepared with the Nano-tRNAseq vs. mRNAe protocols. Significantly differentially abundant tRNAs between sample preparation methods were classified at $p \leq 0.05$, $q \leq 0.05$, and $\log_2$(fold change) ≥ 2.

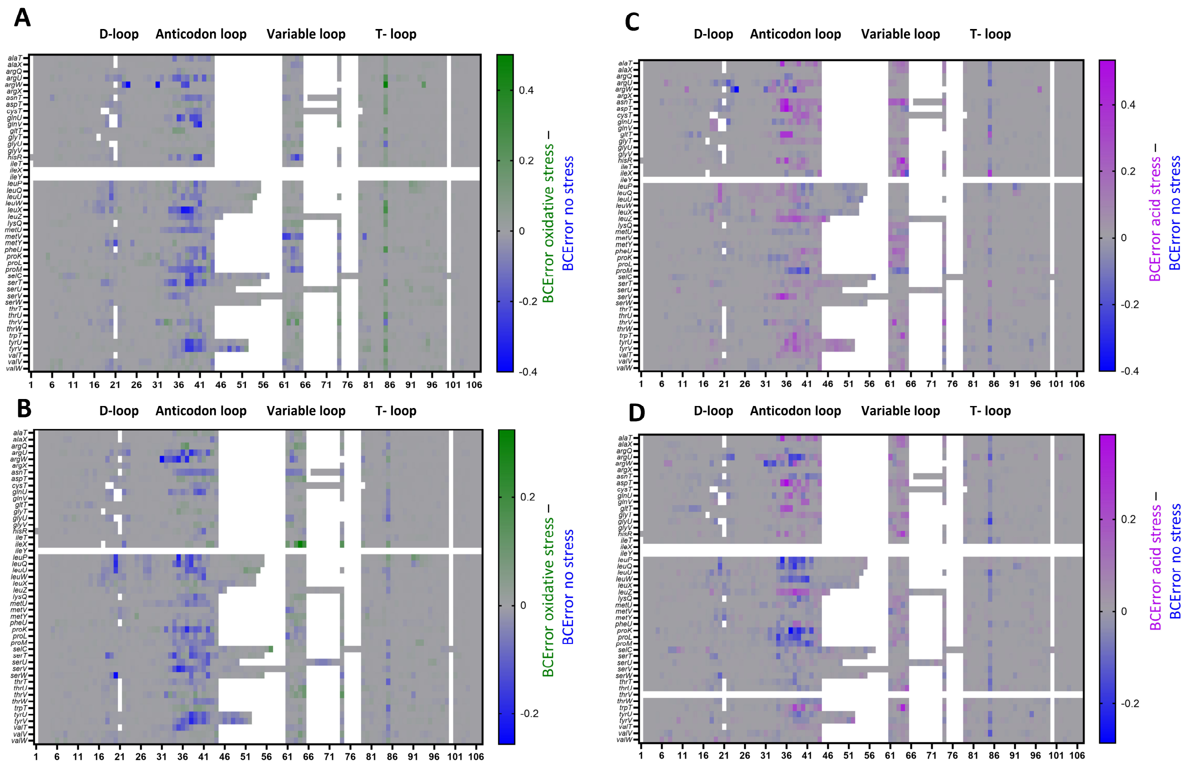

Supplementary Figure 6. Single-sample changes in uncharged *E. coli* pre-tRNA modification abundance during the early stress response. Pre-tRNA abundance in biological replicates one (upper) and two (lower) of the (**A, B**) oxidative and **(C, D)** acid stress samples. Pre-tRNA was prepared and sequenced with the mRNAe protocol; modification levels were determined with BCError. Values for each pre-tRNA position were calculated as $BCError_{Stress}$ – $BCError_{No\ stress}$.

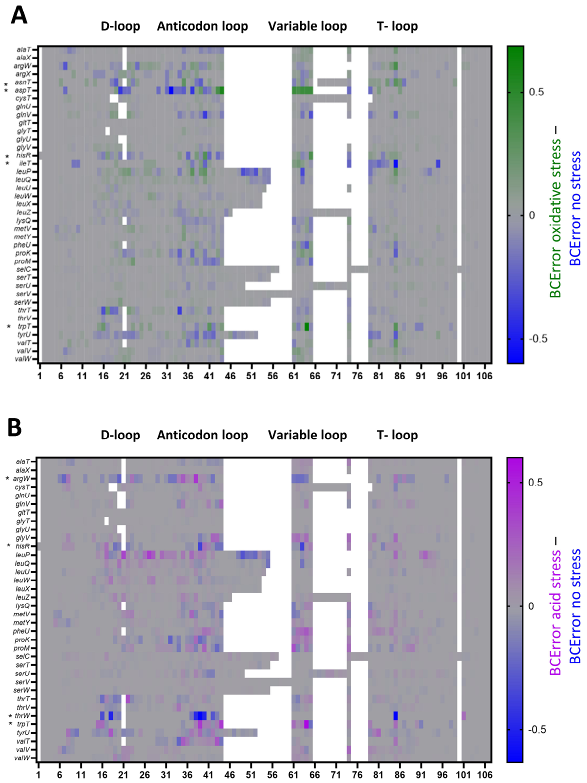

Supplementary Figure 7. Average changes in *E. coli* tRNA modification abundance during the early stress response. (**A, B**) tRNA modification abundance levels in (**A**) oxidative-stress and (**B**) acid-stress samples prepared and sequenced with the Nano-tRNAseq protocol; modification levels were determined with BCError. Values for each tRNA position were calculated as $\text{BCError}_{\text{Stress}} - \text{BCError}_{\text{No stress}}$. Data are shown as the average of two biological replicates; asterisks indicate tRNAs that were present in only one biological replicate. tRNAs were classified as detected at a read depth of ≥ 5.

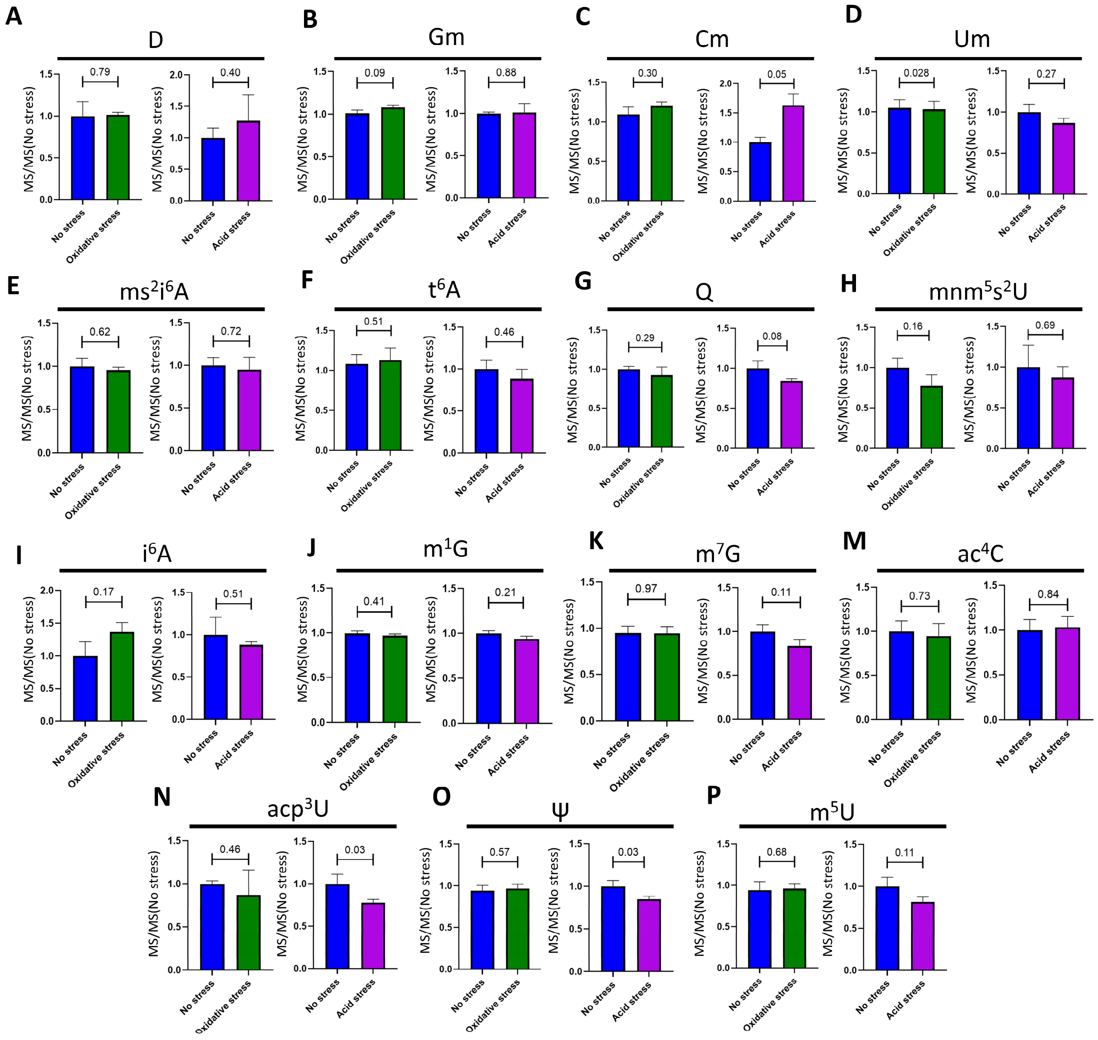

Supplementary Figure 8. Mass spectrometric measurements of modifications in *E. coli* tRNAs. (**A–P**) MS-based quantification of selected modifications in tRNA samples. Measurements were generated from purified RNAs of fewer than 200 nt in size. Modification abundance in each sample type was normalized to abundance in a no-stress control ($MS/MS_{(No\ stress)}$). *p*-values calculated with ratio paired Student's *t*-test. Differences were considered statistically significant at a threshold of $p \leq 0.05$.

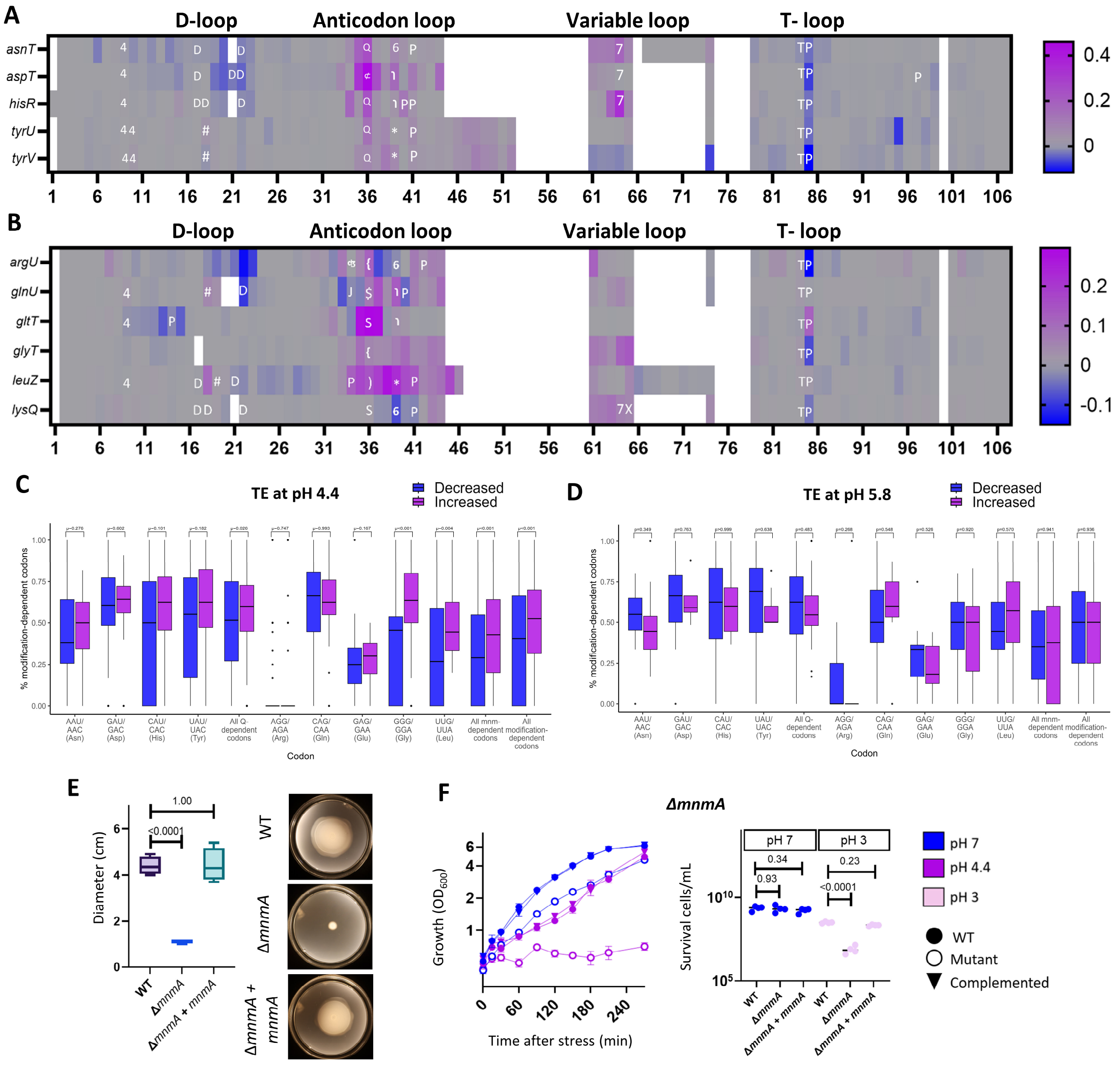

Supplementary Figure 9. Δ*mnmA* mutants show similar phenotypes as Δ*mnmE* and Δ*tgt* mutants. (A, B) Changes in the abundance of (**A**) Q- and (**B**) Mnm-pathway modifications in pre-tRNAs from mRNAe samples. Values were calculated as $BCError_{Acid\ stress}$ – $BCError_{No\ stress}$ for all positions across pre-tRNAs containing Q or Mnm modifications. Data are shown as the average of two biological replicates. Symbols associated with the modification types are consistent with those used in the MODOMICS database (14): ʤ, 2-thiocytidine (s^{2}C) 4, 4-thiouridine (s^{4}U); D, dihydrouridine (D); #, 2′-*O*-methylguanosine (Gm); ⊄, glutamyl-queuosine (gluQ); Q, queuosine (Q); *, 2-methylthio-*N*^{6}-isopentenyladenosine (ms^{2}i^{6}A);), 5-carboxymethylaminomethyl-2′-*O*-methyluridine (cmnm^{5}Um); $, 5-carboxymethylaminomethyl-2-thiouridine (cmnm^{5}s^{2}U); S, 5-methylaminomethyl-2-thiouridine (mnm^{5}s^{2}U); {, 5-methylaminomethyluridine (mnm^{5}U); 6, *N*^{6}-threonylcarbamoyladenosine (t^{6}A); P, pseudouridine (P); T, 5-methyluridine (m^{5}U); ɿ, 2-methyladenosine (m^{2}A); 7, 7-methylguanosine (m^{7}G); X, 3-(3-amino-3-carboxypropyl)uridine (acp^{3}U); J, 2′-*O*-methyluridine (Um). (**C, D**) Proportion of codons requiring modifications synthesized via the Q or Mnm pathways for translation in transcripts having

significantly increased or decreased translational efficiency at (**C**) pH 4.4 or (**D**) pH 5.8 compared to neutral pH according to previously published data (5). (**E**) Swimming motility of Δ*mnmA* mutant and WT *E. coli*. Data are presented as the average of four biological replicates. *p*-values calculated with one-way ANOVA and post-hoc Dunnett's multiple comparisons test. Differences were considered statistically significant at a threshold of $p \leq 0.05$. (**F**) Left, phenotypic analysis of WT and Δ*mnmA* mutants under no-stress and acid-stress (pH 4.4) conditions. Right, survival of WT and Δ*mnmA* mutants under acid stress (pH 3). Stress was induced by the addition of HCl to cultures at OD_{600} = 0.5, then cells were monitored for 4.5 h. Differences in survival were assessed with two-way ANOVA and post-hoc Dunnett's multiple comparisons test and considered statistically significant at a threshold of $p \leq 0.05$.

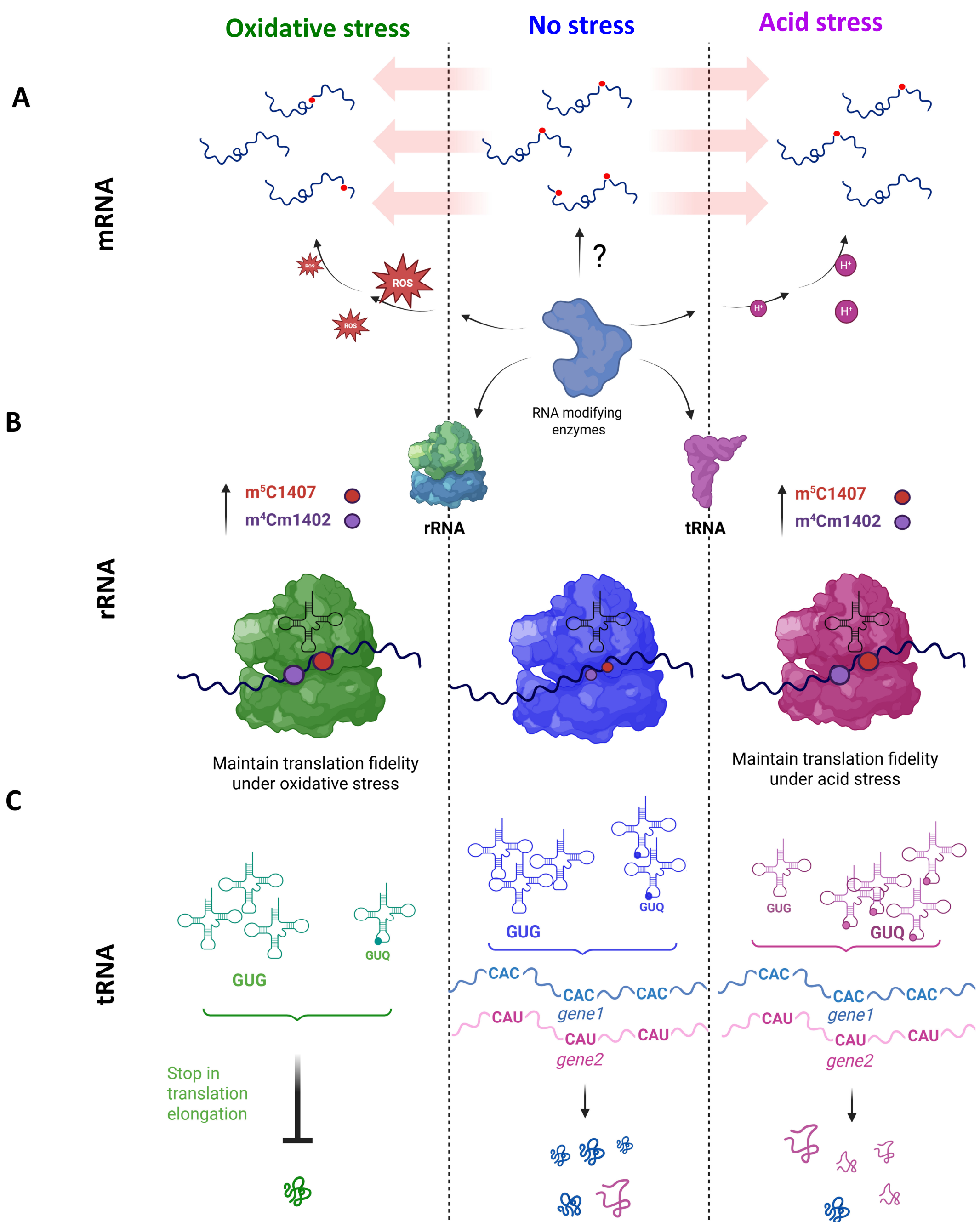

Supplementary Figure. 10. Working model of epitranscriptomic regulation under stress conditions in *E. coli*. (**A**–**C**), Proposed models of (**A**) mRNA, (**B**) rRNA, and (**C**) tRNA modification levels in response to acid- and oxidative-stress conditions. Modification levels in mRNAs are generally low, whereas tRNAs and rRNAs are highly modified. Stress conditions such as acid or oxidative stress alter modification levels in the tRNAs and rRNAs to promote maintenance of essential cellular functions. Increased levels of the 16S rRNA modifications m^{4}Cm1402 and m^{5}C1407 in response to stress conditions may have a protective role, potentially maintaining translational fidelity during stress. Under acid stress, tRNA modifications may contribute to stress adaptation by enhancing the translation efficiency of stress-response proteins, independent of changes in RNA abundance. Images created in BioRender (**A**, https://BioRender.com/6ma8qus; **B**, https://BioRender.com/icv2j3p; **C**, https://BioRender.com/o9lk4xp).
