# Supplementary material for: Epitranscriptomic control of stress adaptations in Escherichia coli
Source: Nucleic Acids Res. 2026 Feb 2;54(3):gkag042. doi: 10.1093/nar/gkag042 (PMC12862378; doi:10.1093/nar/gkag042)
Supplement: gkag042_Supplemental_Files [file gkag042_supplemental_files.zip › Supplementary table legends (clean copy). .docx]

**Supplementary Table S1. Oligonucleotides used in this study.**

**Supplementary Table S2. Mass transitions used in mass spectrometry analyses.**

**Supplementary Table S3. Differential gene expression analysis with direct RNA sequencing data.** Differential expression analysis was conducted using DESeq2. Raw gene-level transcript counts were calculated with Salmon. To minimize noise from lowly expressed genes, only those with at least five reads in each sample were retained for further analyses. Column descriptions are shown in the first tab of the spreadsheet. The presence of putative modifications associated with each gene is indicated. Data are shown separately for acid-stress and oxidative-stress samples.

**Supplementary Table S4. Putative modification sites in mRNA and ncRNA of no-stress, acid-stress, and oxidative-stress samples.** ΔBCError refers to the basecalling error frequency (BCError) at each position in the indicated sample with the BCError of the combined IVT sample at the same position subtracted. For regions (as opposed to single-nt sites) containing a putative modification, ΔBCError values are given for the site in that region with the highest BCError value.

**Supplementary Table S5. Hierarchical KEGG annotations used for functional annotation analyses.** The hierarchical classification scheme for *E. coli* used by Proteomaps (51) was expanded to include an additional ~1000 genes with annotations present in the KEGG database.

**Supplementary Table S6. Relative abundance of Q- and Mnm-pathway dependent codons by transcript.** The tab “Codons” shows the analyzed sets of modification-dependent and -independent codons. “400 most abundant” and “403 least abundant” show the ~400 transcripts with the highest and lowest proportions of Q- and Mnm-dependent codons, respectively.

**Supplementary Table S7. Basic statistics from ONT sequencing of no-stress, acid-stress, oxidative-stress, and IVT control samples.**
